# Evaluation of pliable bioresorbable, elastomeric aortic valve prostheses in sheep during 12 months post implantation

**DOI:** 10.1038/s42003-023-05533-3

**Published:** 2023-11-14

**Authors:** Annemijn Vis, Bente J. de Kort, Wojciech Szymczyk, Jan Willem van Rijswijk, Sylvia Dekker, Rob Driessen, Niels Wijkstra, Paul F. Gründeman, Hans W. M. Niessen, Henk M. Janssen, Serge H. M. Söntjens, Patricia Y. W. Dankers, Anthal I. P. M. Smits, Carlijn V. C. Bouten, Jolanda Kluin

**Affiliations:** 1 Department of Cardiothoracic Surgery, Amsterdam University Medical Centers location University of Amsterdam, Amsterdam, The Netherlands; 2 Department of Biomedical Engineering, Eindhoven University of Technology, Eindhoven, The Netherlands; 3 Institute for Complex Molecular Systems (ICMS), Eindhoven University of Technology, Eindhoven, The Netherlands; 4 Department of Cardiology, Amsterdam University Medical Centers location Vrije Universiteit Amsterdam, Amsterdam, The Netherlands; 5 Department of Cardiothoracic Surgery, University Medical Center Utrecht, Utrecht, The Netherlands; 6 Department of Pathology, Amsterdam University Medical Centers location Vrije Universiteit Amsterdam, Amsterdam, The Netherlands; 7 SyMO-Chem BV, Eindhoven, The Netherlands; 8 Department of Cardiothoracic Surgery, Erasmus MC, Rotterdam, The Netherlands

**Keywords:** Tissue engineering, Preclinical research, Valvular disease

## Abstract

Pliable microfibrous, bioresorbable elastomeric heart valve prostheses are investigated in search of sustainable heart valve replacement. These cell-free implants recruit cells and trigger tissue formation on the valves in situ. Our aim is to investigate the behaviour of these heart valve prostheses when exposed to the high-pressure circulation. We conducted a 12-month follow-up study in sheep to evaluate the in vivo functionality and neo-tissue formation of these valves in the aortic position. All valves remained free from endocarditis, thrombotic complications and macroscopic calcifications. Cell colonisation in the leaflets was mainly restricted to the hinge area, while resorption of synthetic fibers was limited. Most valves were pliable and structurally intact (10/15), however, other valves (5/15) showed cusp thickening, retraction or holes in the leaflets. Further research is needed to assess whether in-situ heart valve tissue engineering in the aortic position is possible or whether non-resorbable synthetic pliable prostheses are preferred.

## Abstract (web summary)

In situ tissue engineered heart valve prostheses implanted in aortic position of an ovine model up to 12 months meet several hallmarks for sustainable heart valves prostheses, but variability in tissue remodelling and valve functionality are seen.

## Introduction

Due to an aging population, the prevalence of valvular heart disease is growing worldwide^1,2^. End stage valvular disease is typically treated by replacement of the valve, with either a mechanical or biological heart valve prosthesis. Both types of prostheses are associated with serious drawbacks, such as thromboembolic complications or calcification-induced limited durability^1–3^. The pressing need for improved heart valve prostheses has stimulated the pursuit of sustainable replacement options. Hallmarks of a sustainable heart valve include that the valve 1) is a living tissue capable of adaptation, 2) has good long-term functionality, 3) is non calcifying, 4) is not prone to infection, 5) is non-thrombogenic and, 6) is non-immunogenic^4^. To date, no heart valve prosthesis has been developed that meets all these properties. In search of the optimal sustainable heart valve, several innovative types of heart valve prostheses have been proposed^5^. In the past two decades, preclinical and clinical studies demonstrated promising results for the use of various leaflet materials, including decellularized allogenic and xenografs^6–8^, in vitro cultured decellularized grafts^9–11^ and degradable synthetic polymers^12–18^. The development of fibrous bioresorbable synthetic heart valves, preferably based on elastomers, is amongst the most novel approaches, potentially capable of meeting all hallmarks, and therefore overcoming the intrinsic limitation of traditional heart valve prostheses^5,12,19^.

Bioresorbable elastomeric valves can be made in a reproducible and scalable fashion, and can be made available “off-the-shelf”. They are cell-free when implanted in the body and their microfibrous structure is designed to trigger regeneration of the valve, directly in situ^5,20^. In situ tissue engineered heart valves rely on endogenous colonisation of the implanted valve prosthesis by host cells, followed by in situ tissue formation and remodelling, while the implanted fibrous synthetic valve (scaffold) is gradually resorbed^20,21^. The balance between neo-tissue formation and graft resorption is of utmost importance for the valve to remain functional, as too fast resorption might induce early graft failure and abundant tissue deposition might induce thickening and eventually retraction (shortening) of the leaflet. If efficient, eventually, a living valve will be created within the body able to adapt to functional demand changes^22,23^. Several research groups have tested fibrous bioresorbable elastomeric heart valves in the pulmonary position in chronic animal trials^10,12,14–16,24^. When implanted in the pulmonary circulation, the valve leaflets are exposed to low pressures and moderate hemodynamic conditions. The results of these chronic preclinical trials are known to be variable. Some, studies report promising results, including good valve functionality and the colonisation of the valve leaflets with cells^12,13^. Other, studies report interleaflet and intervalve variability, including undesired leaflet remodelling, such as shortening and thickening of the valve leaflet that may result in valve regurgitation^12–14,16^. Although the mechanisms underlying this variability have yet to be understood, rapid progress is made in the field, including the development of pulmonary transcatheter approaches^16^ and the first in human implantation of pulmonary elastomeric bioresorbable valves^18^. Therefore, a logical consequent step is to investigate these bioresorbable elastomeric heart valves in the complex hemodynamic environment of the high-pressure circulation. Long term in vivo assessment of bioresorbable elastomeric heart valve prostheses in aortic position has not been carried out before, partly due to the technically difficult surgical procedure on the arrested heart, and partly due to the yet unknown influence of high systemic pressures on tissue formation and remodelling of the heart valve. Here, we investigate the concept of in situ tissue engineering of aortic heart valves in a chronic animal model. We employed bis-urea-modified polycarbonate as the valve material, a state-of-the-art material known for its ability to facilitate in vivo cell colonisation and resorption, evidenced by our previous study with pulmonary valves^13^. We monitored the long-term performance of bioresorbable, pliable elastomeric heart valves in the high-pressure systemic circulation, in terms of functionality, host cells recruitment, in situ neo-tissue formation and scaffold resorption.

## Results

### Surgery and complications

We implanted the tri-leaflet bis-urea-modified polycarbonate (PC-BU) aortic valves in the orthotopic position in twenty female Swifter sheep. Fifteen of twenty animals (75%), (mean weight 67 ± 5 kg and mean age 2.0 ± 0.2year) recovered well from the surgical valve implantation procedure and were included in this analysis (Supplementary Fig. 1). Mean cardiopulmonary bypass (CPB) time was 152 ± 44 min, mean aortic cross clamp time was 77 ± 24 min. We lost five of twenty animals (25%) due to surgical complications unrelated to the valve prosthesis itself. One sheep died on the table due to refractory ventricular fibrillation. A second sheep died on the first post-operative day (POD 1) due to severe aortic regurgitation caused by an aortic valve leaflet that remained in open position. This was caused by one loose fibre of the electrospun valve leaflet scaffold that was caught in a suture probably by one additional haemostatic stitch after the aorta was closed and the echo was made. A third sheep died on POD 1 due to high lactate levels, a complication related to the CPB support. A fourth sheep died on POD 2 due to postoperative haemorrhage. A fifth sheep died on POD 1. This death was likely attributed to the prolonged CPB duration and substantial blood loss (Hb 2.4 mmol/L) caused by extensive efforts undertaken to stop a leak along the aortic suture line. The fifteen surviving animals were randomly appointed to the following follow-up (FU) periods: 1 month (*n* = 2), 3 months (*n* = 3), 6 months (*n* = 3), 9 months (*n* = 3) and 12 months (*n* = 4). One sheep, selected for the 9 months FU group, died 6 months after valve implant. The cause of death could not be identified but did not appear to be valve-related. We have analysed this sheep as part of the 6 months FU group, as valve #6.4 (Supplementary Fig. 1). The post-operative period was complicated in four cases, not leading to death. One sheep required inotropic support with dobutamine (10 mcg/kg/min IV, dobutamine 12.5 mg/ml, Centrafarm, Etten-Leur, Netherlands) during the first night (#1.1). One sheep developed postoperative pleural effusion (#6.4) and one sheep had a cardiac tamponade (#12.4). Both sheep could be treated with a thorax drain. We found pleural effusion and ascites in one sheep during explant, without any clinical signs (#12.2).

### In vivo valve functionality

**Fig. 1:**
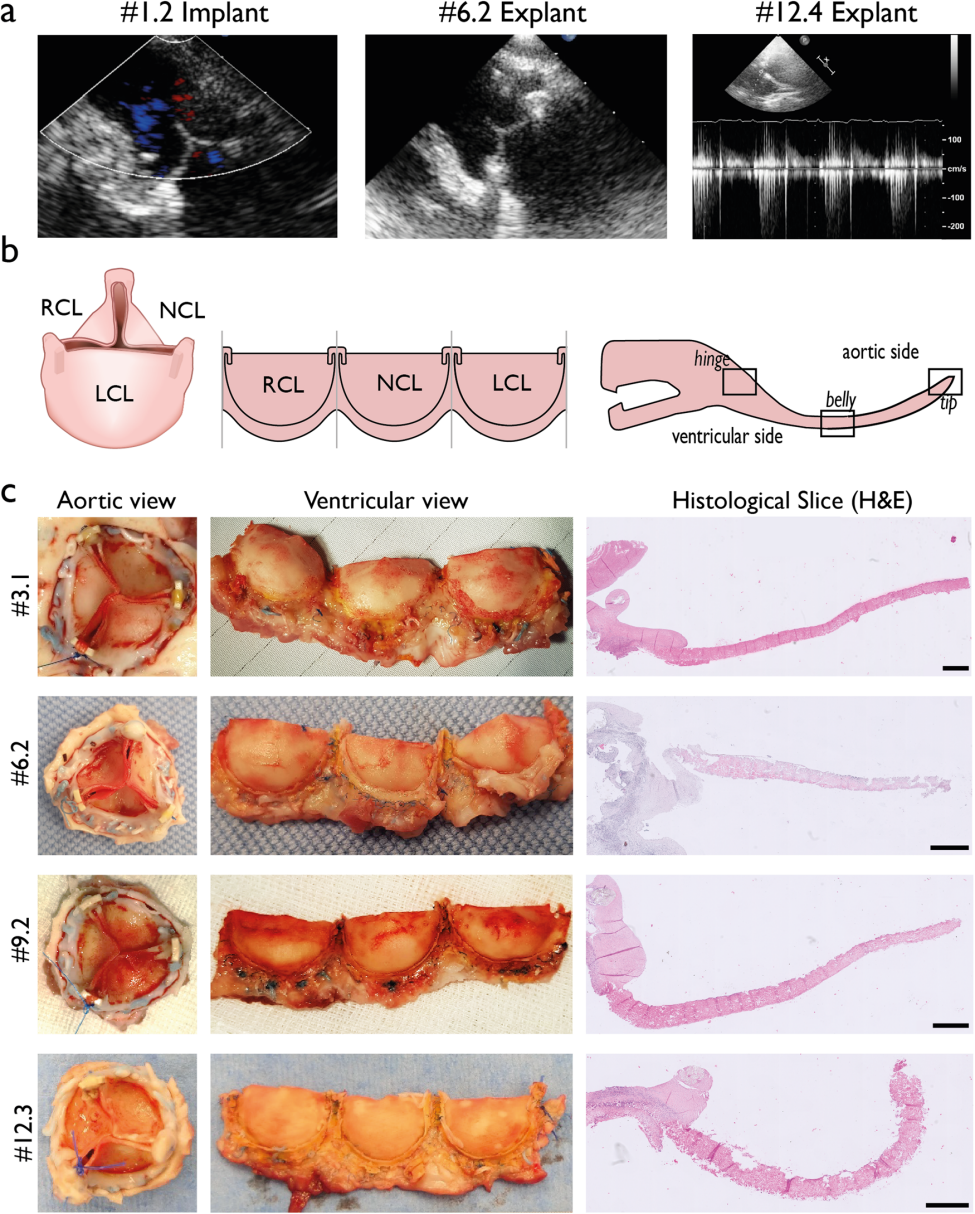
Gross morphology and in vivo functionality. **a** Echocardiographic measurements of representative valves at implantation, and after 6 and 12 months follow up. **b** Schematic orientation of the heart valve prosthesis. RCL right coronary leaflet, NCL non coronary leaflet, LCL left coronary leaflet. **c** macroscopic images of one representative valve for 3, 6, 9 and 12 months follow-up. Left image is the intact valve from aortic view, middle image are the dissected leaflets from ventricular view, right are histological slices of a valve leaflet stained with H&E. Scale bar is 1 mm.

We obtained ultrasound measurements for all sheep during explant (Table 1, Supplementary Table 1, Supplementary Movies 1-3), except for sheep #6.4 due to its sudden death. At explantation, the left ventricular systolic function was normal in all sheep except: #1.1, #9.2, #12.1 (mild abnormal) and #12.2 (severe abnormal). All the valves showed none or mild stenosis on echo, except valve #9.1 (moderate) and valve #12.1 (severe). Invasively measured mean aortic transvalvular peak gradients did not increase over time; 31 ± 4 mmHg at 3 months, 31 ± 4 mmHg at 6 months, 36 ± 14 mmHg at 9 months and 33 ± 14 mmHg at 12 months. This gradient was severely increased in two sheep; #9.1 and #12.1 (Supplementary Table 2). Most valves showed none or mild regurgitation (Fig. 1). Two valves showed moderate regurgitation: #1.1 (paravalvular, non-structural failure) and #9.1 (valvular, structural failure). Three valves showed severe regurgitation, concomitant with a dilated left ventricle: #6.1, #12.1 and #12.2 (structural valve failure).

**Table 1:** In vivo functionality of PC-BU aortic valves.

	1 month *N* = 2	3 months *N* = 3	6 months *N* = 3	9 months *N* = 2	12 months *N* = 4
Valve Insufficiency Grade	Mild: 1 Moderate: 1	None: 1 Mild: 2	Mild: 2 Severe: 1	None: 1 Moderate: 1	Mild: 2 Severe: 2
Valve Stenosis Grade	None: 1 Mild: 1	None: 3	None: 3	None: 1 Moderate: 1	None: 2 Mild: 1 Severe: 1
P_LVOT_ systole, mmHg (mean, SD)	X	120 ± 18	86 ± 14	81 ± 5	95 ± 9
P_LVOT_ diastole, mmHg (mean, SD)	X	107 ± 17	68 ± 18	64 ± 12	68 ± 10
P_LV_ systole, mmHg (mean, SD)	X	150 ± 20	117 ± 13	117 ± 5	128 ± 5
Transvalvular ΔP peak-to-peak gradient, mmHg (mean, SD)	X	31 ± 4	31 ± 4	36 ± 14	33 ± 14

*P* Pressure, *LVOT* left ventricular outflow tract, *SD* standard deviation, *LV* left ventricle, *ΔP* P_LV_-P_LVOT_.

### Gross explant evaluation

**Fig. 2:**
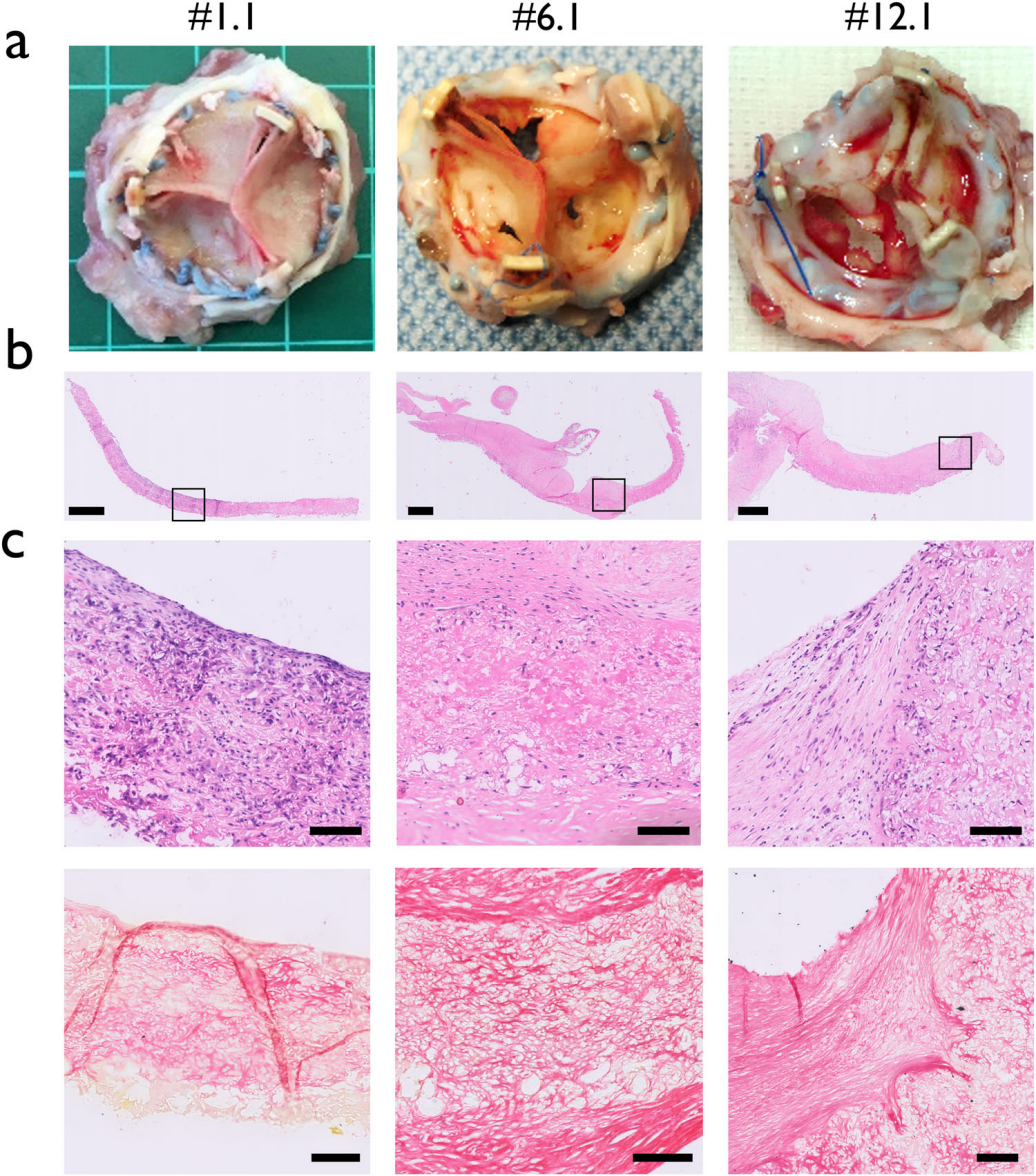
Variability in valve morphology and histology. **a** Macroscopic images of valves #1.1, #6.1 and #12.1. Valve #1.1 is a intact with thin and pliable leaflets. Valves #6.1 and #12.1 represent maladaptive remodelling processes, such as leaflet retraction and thickening (#12.1), leaflet degradation (#6.1 and #12.1), and white coloured neotissue deposition (#6.1). **b** Entire valve leaflet of valves #1.1, 6.1 and 12.1 with H&E staining, scale bar 1 mm. **c** Top row indicates the belly region with H&E staining, scale bar 100 µm. Histological slides match the valves as shown in A. Bottom row indicates belly regions of the respective valves stained with Picrosirius Red staining, collagen appears red. Scale bar is 100 µm. The scaffold of valve #1.1 is thin and abundantly populated with host cells, especially in the belly region. The scaffold of valves #6.1 and 12.1# are covered with a cell-rich thick layer of neo tissue on the aortic side, consisting of mature collagen.

Macroscopically, pliable valve leaflets without signs of (non-) structural valve deterioration have been observed in 10 sheep (67%) up to 12 months after valve implantation (Fig. 1). However, deterioration of one or more leaflets was observed in five valves (33%). The right coronary cusps of two valves (13%), #9.1 and #12.1, were retracted and thickened (Fig. 2a, Supplementary Fig. 2). Partial degradation of one of more leaflets resulting in holes in the leaflets, was observed in four valves (27%) without any signs of thrombus formation, #6.1, #9.1, #12.1, #12.2 (Fig. 2a, Supplementary Fig. 2). One valve (7%) showed a tear in one leaflet, close to the polyether ether ketone (PEEK) ring #12.4 (Supplementary Figure 2). Apart from valve deterioration, some other irregularities have been observed. In valve #3.3, one leaflet was not properly attached to the strut and slipped away, a flaw in the design of the valve. No macroscopic calcification was visible, except for a small calcific area detected at the strut in valve #12.3, but not at any leaflets. White coloured neotissue regions were observed on some valve leaflets of valves #6.1, #6.3, #9.1 and #12.1 (Fig. 2a, Supplementary Fig. 2).

### Calcification assessment

**Fig. 3:**
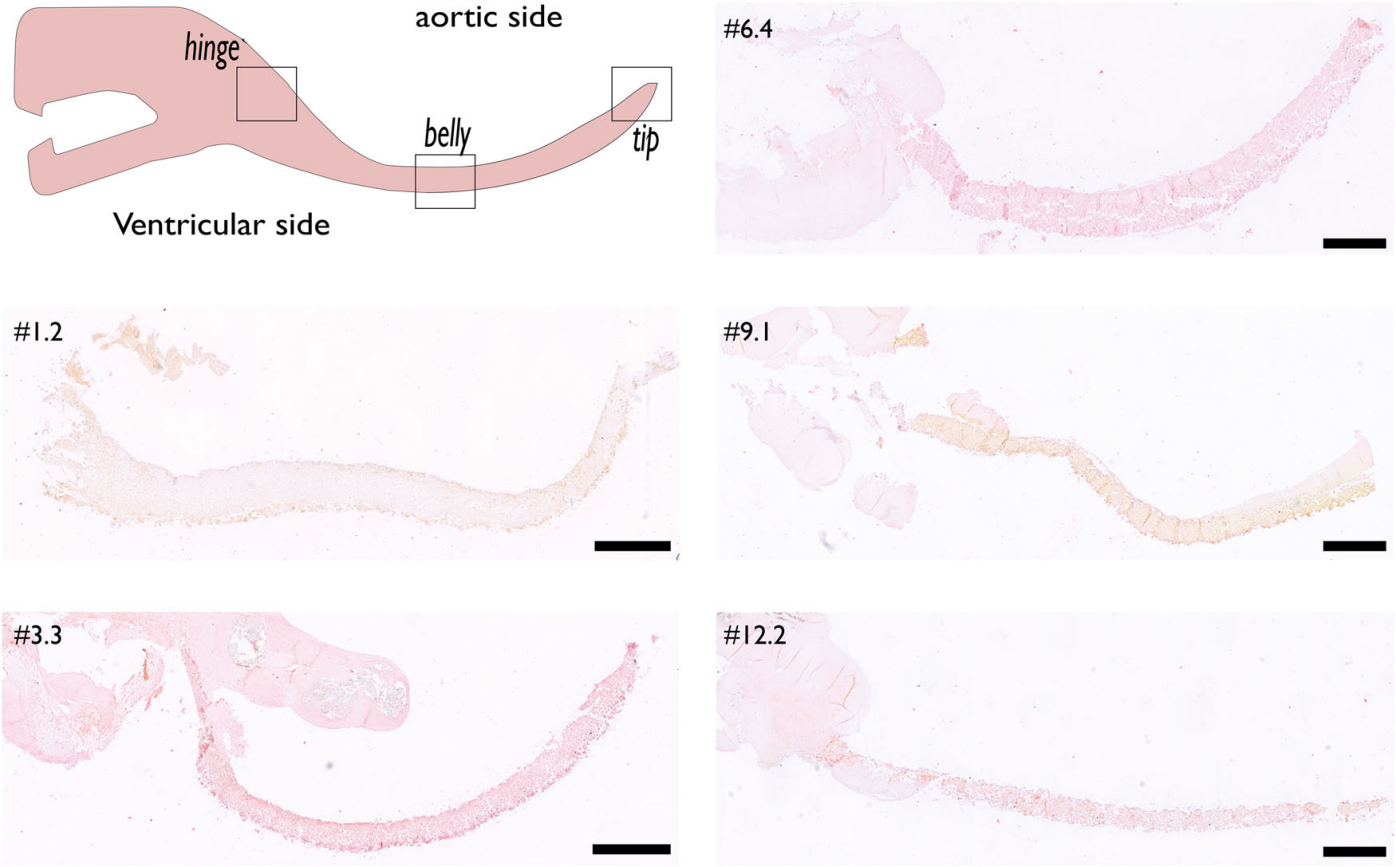
Calcification assessment. Representative slides depicting a whole valve leaflet from representative valves of each follow up period. Slides are stained with Alizarin Red staining, in which calcium deposits appear as bright red and non-calcified tissue as light pink. Pathological calcification was absent in any of the valves. Scale bar is 1 mm.

Throughout the 12 months follow-up period, the majority of valves remained free from any histological calcification (Fig. 3). Based on the quantitative calcification score, most valves (73%) were classified as non-calcified, while four valves (27%) were graded as mildly calcified. The only calcific sites that were observed in these valves were micro calcifications, with the largest diameter being 66 µm, which is not considered clinically relevant^25^ (Supplementary Fig. 3).

### In situ colonisation of valves by host cells

**Fig. 4:**
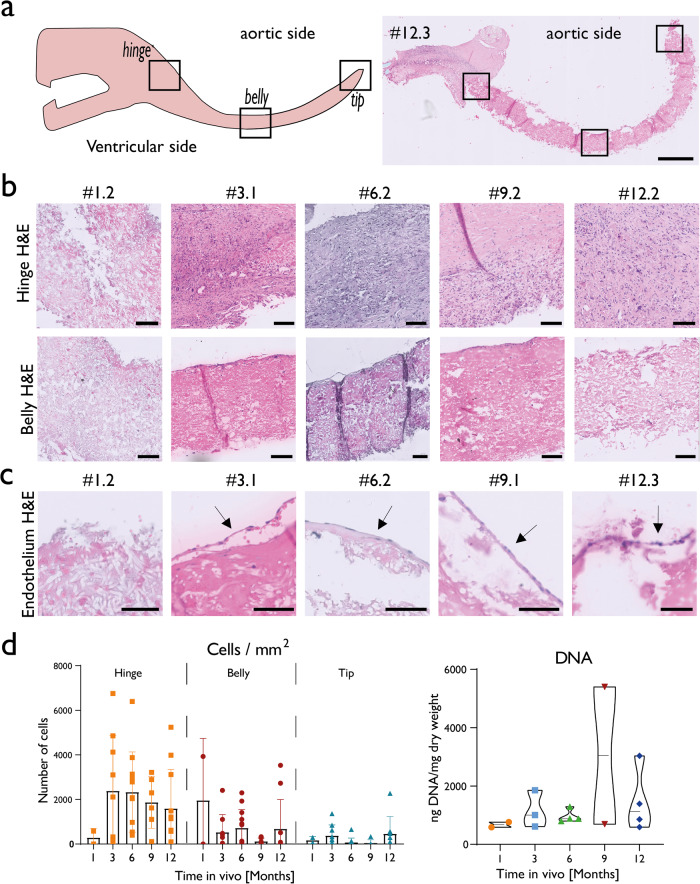
In situ colonisation of valves by host cells. **a** Left; schematic leaflet with regions of interest. Right; one representative valve leaflet stained with H&E. Scale bar 1 mm. **b** Belly and hinge regions for one representative valve for each time point. Colours indicate nuclei (dark purple) and tissue (pink), Scale bar is 100 µm. Cells infiltrate abundantly in hinge regions, while belly regions are free from cells. **c** Endothelium in belly regions indicated with an arrow. Scale bar is 50 µm. **d** Left: Semi-quantification of cells present per mm² in and on the scaffold in regions of interest counted in H&E staining. Error bars are standard deviations. Bar charts are means. The hinge regions of the valves were colonised by host cells from 3 months onwards. Right: DNA quantification, normalised to valve dry weight, measured for half a valve. Box plot present maximum and minimum values, middle horizontal bar represents the mean. Limited amount of DNA was measured with exception for one 9 month and one 12 month valve, ea. #9.1 and #12.1.

All valves remained free from active inflammation and infectious endocarditis. Generally, a limited number of cells infiltrated the graft material over time as indicated by valve deoxyribonucleic acid (DNA) measurement and histology (Fig. 4a, Supplementary Fig. 2). Only in the hinge region numerous cells infiltrated in the porous microstructure of the scaffold of all valves, from three months onwards. The belly and tip regions of the scaffold remained sparsely- or unpopulated by cells throughout the entire follow-up period. In unpopulated scaffold regions, an acellular amorphous mass was observed in between the scaffold fibres (Fig. 4b). Cell count inside the scaffold indicated a trend in cellularity in the hinge region, where cell colonisation was highest at 3 and 6 months, after which it gradually decreased at 9 and 12 months (Fig. 4d). Exceptional cases showed that cells infiltrated into the leaflets of the valves. In one leaflet of valve #1.1, cells were present in the scaffold in the belly region. Additionally, in thickened leaflets, such as #12.1, numerous cells were present in the neo tissue and in the scaffold (Fig. 2b, c). Systematic assessment of endothelialisation was hampered by difficulties in sectioning due to the presence of the synthetic scaffold materials. When observed, endothelium was predominantly present on the hinge and belly regions of the aortic side of the valve tissue or on the neo tissue layer. In contrast, on the ventricular surface the scaffold remained largely exposed and bare (Fig. 4c).

### Neo tissue formation and matrix deposition

**Fig. 5:**
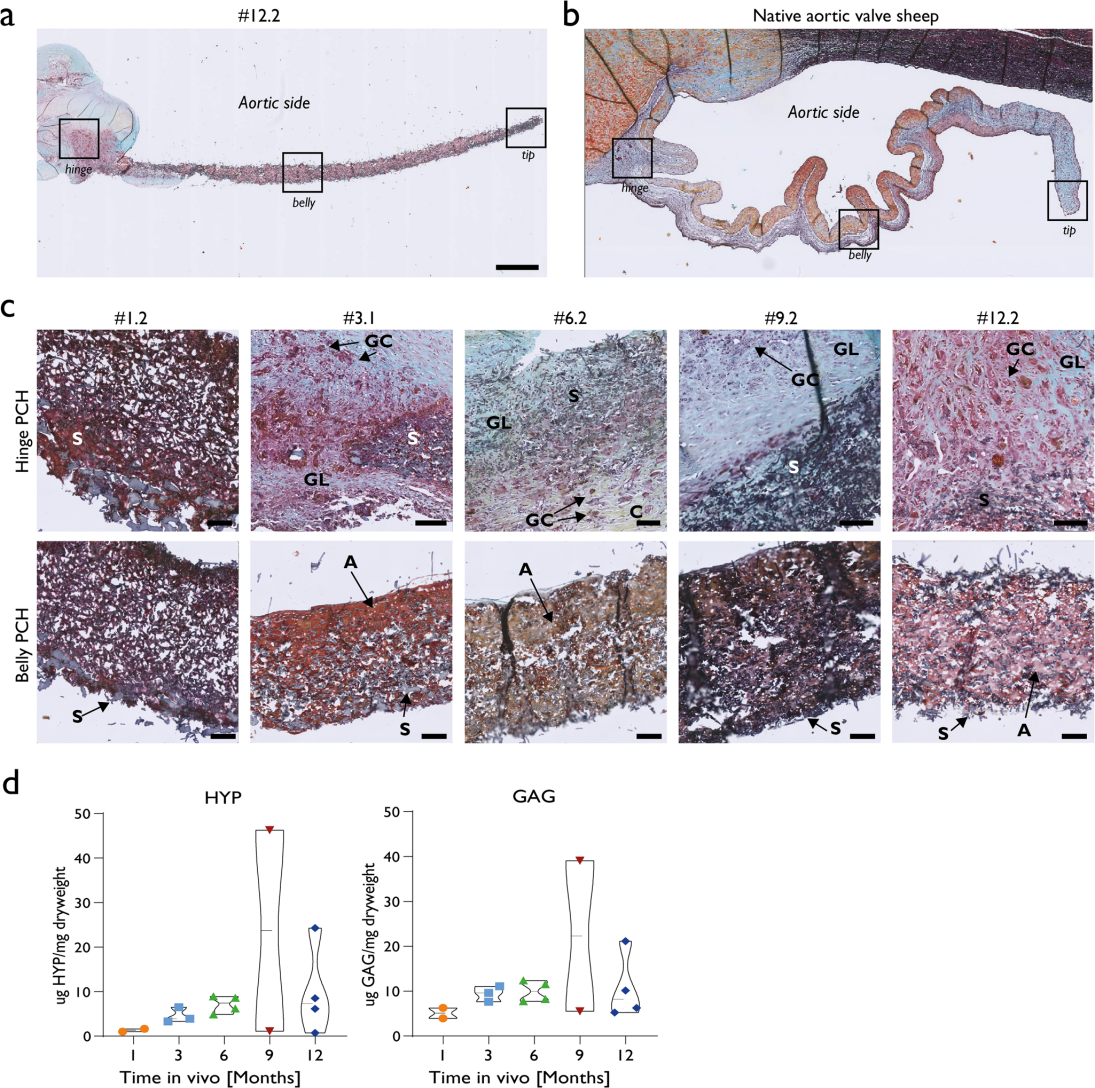
General composition of deposited neotissue. **a** Representative longitudinal section of a 12 month explant and (**b**) a native sheep aortic valve both stained with Movat Pentachrome. Colours indicate glycosaminoglycans (blue), collagen (yellow), cells or muscle (purple/pink), elastic fibres (black), elastomeric scaffold (dark blue). Scale bar 1 mm (**c**) Hinge and belly regions of representative valves of each follow up time are depicted with Movat Pentachrome staining. Here, active regions with giant cells (indicated by GC), graft resorption (scaffold indicated with S) and local tissue deposition consisting of glycosaminoglycans (indicated by GL) and collagen (indicated by C) were present. In the belly region, a brown dense a-cellular amorphous mass was detected between scaffold fibres, (indicated by A). Scale bars 100 µm. **d** Biochemical quantification of HYP, as a measurement of collagen, GAG as tissue deposition in de leaflets. Box plot present maximum and minimum values, middle horizontal bar represents the mean. In 2 leaflets, #9.1 and #12.1, higher HYP and GAG contents were measured.

Most valves remained free from any neo-tissue formation adhering onto the scaffold in belly and tip regions, resulting in smooth and thin valve leaflets, up to 12 months follow-up. Movat Pentachrome staining indicated that generally only in the hinge regions, sections with cell colonisation (red staining), giant cell formation (indicated by GC) and matrix formation were present, the latter consisting of glycosaminoglycans (blue staining, indicated by GL) and collagen (yellow staining, indicated by C) (Fig. 5). Five valves were colonised with cells in the belly and tip regions. Valve #1.1 was colonised with cells and was also thin and pliable. The other four valves were also colonised with cells, but showed a thick layer of neo-tissue as well as partial degradation of one or more leaflets (#6.1, #6.3, #9.1 and #12.1) (Fig. 2).

**Fig. 6:**
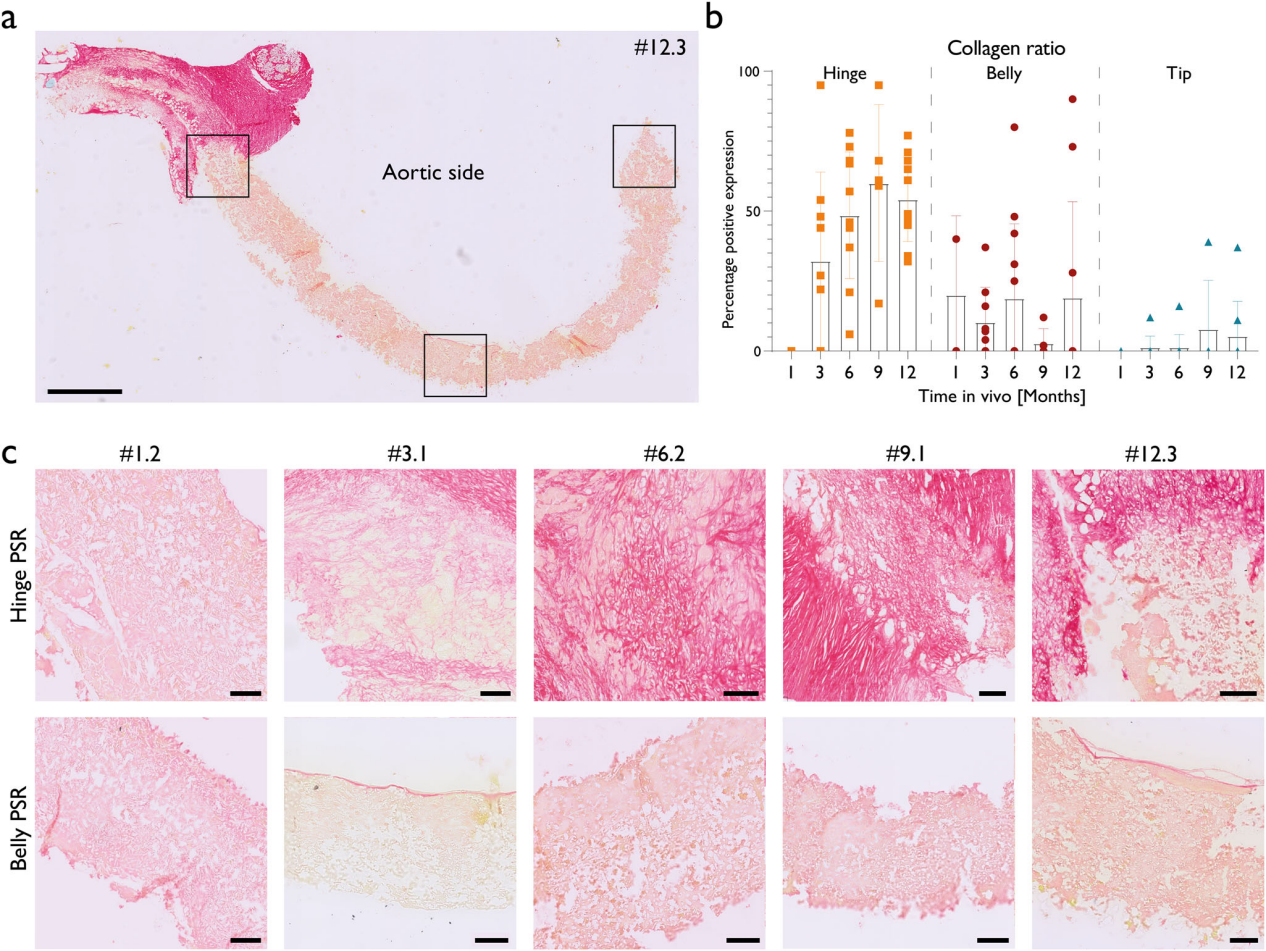
Collagen deposition and structure. **a** Picrosirius Red staining on a representative longitudinal section of an entire valve leaflet of a 12-month explant. Collagen is depicted in red, scaffold in light yellow. Scale bar is 1 mm. **b** Semi-quantification of collagen formation. For each region of interest, the area covered by collagen fibres is divided by the total area. Error bars are standard deviations. Bar charts are means. The deposition of collagen in and onto the scaffold is mainly observed in the hinge region. **c** Belly and hinge regions of representative valves are depicted for each time period, stained with Picrosirius Red staining. Scale bar is 100 µm. The deposition of collagen is generally observed in hinge regions but not observed in belly regions.

The deposition of mature, fibrous collagen in and onto the scaffold in the hinge region increased steadily over time, stabilising at 9 months after valve implantation (Fig. 6b). Generally, there was no collagen deposition observed in the belly and tip regions at all time points (Fig. 6a, c, Supplementary Fig. 4). However, in some exceptions, mature collagen deposition inside and onto the scaffold at the belly and tip regions of the valve leaflets were observed. This correlated with cell-rich regions, such as seen in valves #1.1, #6.1, #6.3, #9.1 and #12.1. (Fig. 2b, c). Similar results were obtained with biochemical quantification of hydroxyproline, a measure for collagen, and glycosaminoglycans. Both cell-deposited proteins increased slightly with increasing implantation time. Only one 9-month valve and one 12-month valve, #9.1 and #12.1 respectively, showed higher amount of collagen and glycosaminoglycans in the leaflet (Fig. 5). Mature elastic fibres (black staining) were not observed in the scaffolds or neo-tissue, in neither region at neither follow up period (Fig. 5).

### Scaffold resorption

**Fig. 7:**
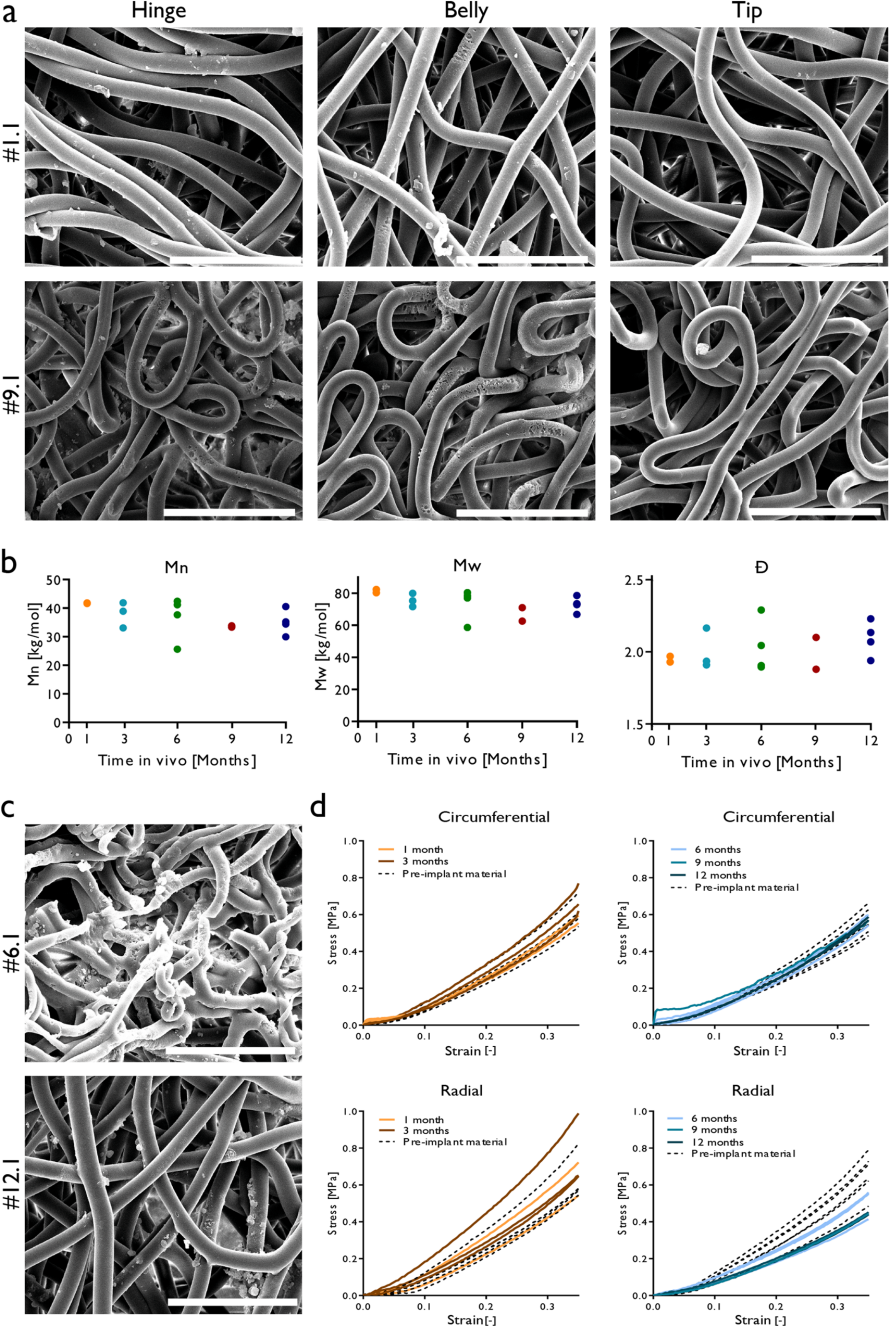
Scaffold resorption and mechanical behaviour. **a** Representative scanning electron images of the hinge, belly and tip regions of a 1- and 9-month explants. Minimal fibre resorption was present in the hinge and belly region only. Scale bars 30 µm. **b** GPC measurements indicating little to no degradation in the remaining scaffold material. Most degradation was detected in valve #6.1. **c** Regionally, fibres were resorbed more extensively in valves #6.1 and #12.3 as indicated by the SEM images. Scale bars 30 µm. **d** Mechanical testing indicated similar mechanical behaviour of the synthetic material before implantation and after explantation of the valves. We observe a trend in which the stiffness of the valves is slightly higher in the 1- and 3-month explants and slightly lower in the longer follow up times compared to the pre-implant synthetic material.

After explantation and decellularization, Scanning electron microscopy (SEM) and gel permeation chromatography (GPC) molecular weight measurements (Fig. 7a, b) indicated little to no scaffold fibre resorption of the present synthetic material and minimal decrease in molecular weight of the polymer for all scaffolds implanted up to 12 months. Most synthetic fibres were still intact with minimal reduction in diameter (Supplementary Fig. 5), however when resorption was present, it occurred mostly on fibres in the hinge and belly regions. Even scaffolds with cell infiltration, e.g., #12.1, showed limited resorption (Fig. 7c). In two exceptional cases, #6.1 and #12.3, more pronounced graft resorption was present regionally. In valve #6.1, the lowest polymer molecular weight was measured. Histologically, synthetic fibres were visible up to 12 months throughout the leaflet, however most apparent in belly and tip regions. In the more cellularized hinge regions the synthetic fibres became less apparent especially after longer follow-up durations. Regions of active resorption of the scaffold fibres by giant cells was seen in all valves from 3 months onwards (Supplementary Fig. 6). Particularly at the hinge regions of the valves, resorption of the PC-BU scaffold took place. Limited decrease in polymer molecular weight, fibre resorption and collagen deposition also correlated to a minimal change in mechanical behaviour of the explanted leaflets compared to the pre-implant synthetic material both in circumferential and radial direction. We observed a trend in which the stiffness of the valves was slightly higher in the 1- and 3-month explants and slightly lower in the longer follow up times compared to the pre-implant synthetic material (Fig. 7d).

### Thrombosis assessment

We conducted histological analysis to assess the putative presence of thrombi in arteries of the liver, spleen, kidney and lung of all sheep except one (*n* = 14). The sheep that was found dead in the stable after 6 months of follow-up could not be analysed further due to autolysis of the tissue and was therefore excluded (#6.4). No recent or older thrombi were found in the examined organs. Also no major pathological changes of the organs were found, except for a non-specific acute inflammation of the liver but without foreign body granulomas (Supplementary Fig. 7).

## Discussion

This study is the first to test the remodelling of biodegradable elastomeric valve prostheses in the aortic position in a preclinical large animal model. We demonstrate that the bioresorbable elastomeric valves meet several of the hallmarks for sustainable valve replacements as they generally have pliable and functional leaflets and remain free of infectious endocarditis, thrombotic events and macroscopic calcifications at 12 months after implantation. However, we also observed intervalve and interleaflet variabilities in tissue outcomes, including maladaptive remodelling of some valve leaflets that impaired valve functionality. Generally, limited cell infiltration and consequently limited progression in tissue deposition and scaffold resorption was detected, indicating slow or minimal remodelling towards a living valve. We conclude that successful outcomes obtained with identical valves in the pulmonary position^13^ cannot be 1-to-1 translated to the aortic position.

The search for a true sustainable heart valve replacement is ongoing. Although bioprosthetic valves are frequently employed for implantation in the clinic, their sustainability is limited to 10–15 years due to poor durability and early graft calcification^19^. Early degenerative failure of bioprosthetic valves is even more profound in paediatric patients, making bioprosthetic valves unsuitable for children with congenital heart disease^26^. Animal trials in which commercial bioprosthetic valves are implanted in the high-pressure circulation show calcifications after 6 to 8 months of implantation^27–29^. The use of decellularized homografts is currently very limited due to the shortage of human organ donors^19^. Other approaches such as decellularized xenografts or decellularized in vitro cultured matrices have been implanted in the aortic position during pre-clinical animal trials. Although generally good acute functionality was reported^30,31^, long-term outcomes were more variable. For decellularized porcine and rabbit valved conduits as well as for valves made from fibrin-based decellularized in vitro cultured matrices, good hemodynamic performance with host cell infiltration and tissue remodelling was reported for pre-clinical implantation up to 6 months^32–34^. On the other hand, clinical trials using decellularized xenografts valved conduits composed of porcine small intestinal submucosa (SIS) reported no advantage of this material over polytetrafluoroethylene^30^. One clinical trial using decellularized porcine heart valves even reported sudden structural failure resulting in the death of 3 of 4 paediatric patients^35^. In other studies, in which porcine SIS was used as a patch for aortic valve repair, reoperations were required after 1.5 years due to high inflammatory infiltration causing structural changes and after 4 years due to high incidences of calcification, fibrosis and retraction^36,37^. Clinical research with these materials should therefore be performed with caution^19^.

The use of bioresorbable elastomeric scaffolds is an emerging and promising concept in search for sustainable heart valve prostheses. First, the remodelling capacity of the elastomeric bioresorbable heart valves could provide a life-long sustainable heart valve prosthesis, avoiding the need for strict antithrombotic regimens. Next, the leaflets these scaffolds are thin and pliable, making them suitable for future transcatheter applications^38^. Contrary to other approaches that aim to develop sustainable heart valve prosthesis, such as decellularized homografts and bioprosthetic valves, bioresorbable elastomeric scaffolds are fabricated from off-the-shelf available materials, making these valves economically attractive, scalable in production and independent from the availability of any donor materials. A biodegradable elastomeric scaffold deployed as a transcatheter aortic valve implantation may provide a very low-cost therapy for rheumatic valvular disease, which is greatly demanded in developing countries^19,38^. Bioresorbable elastomeric heart valves as pulmonary valve prostheses have been evaluated in pre-clinical studies and in one ongoing clinical trial^10,12–14,16–18^. They are in general extensively colonised by host cells and tissue remodelling progresses from the hinge region towards the tip of the leaflet, however maladaptive remodelling of several valve (leaflets) has also been reported. No chronic in vivo trials have been conducted in which bioresorbable elastomeric heart valves are exposed to the harsh hemodynamic conditions of the systemic circulation.

For the present study, we used the same scaffold design, elastomeric material, and fabrication method as we earlier successfully applied as pulmonary valves implants^13^. In contrast to the pulmonary implants^13^, the valves implanted in the aortic position, showed limited cell infiltration, which was mostly restricted to the hinge region throughout the entire follow-up period. We have seen that proteins that originate from the blood, such as fibrin, were deposited between the scaffold fibres and formed a dense amorphous mass there. Despite the absence of cells, most valves were thin and pliable and maintained their mechanical strength (Fig. 7), which is an important hallmark for sustainable valve replacement. The limited resorption of scaffold fibres underlines the notion that the degradation of synthetic material is highly cell dependent. We further found that endothelialisation of the valve leaflets was incomplete and endothelium was only present at subsections, predominantly at the aortic side, while at the ventricular side, the synthetic fibres were mainly uncovered by endothelium. Though, the extent of endothelialisation remains speculative due to possible damage of the endothelial layer during sectioning. The presence of a healthy native endothelium is considered essential for growing a haemocompatible blood-scaffold interface and thus for preventing thrombotic events and long-term functionality of the valve prosthesis. In this study, in which only carbasalate calcium was given as anticoagulation therapy in the first 90 days, no thrombogenic events were seen. The potential of the formation of an endothelial layer on a purely synthetic heart valve has great clinical benefits, and therefore deserves to be investigated further in future trials. The limited cell infiltration and consequently limited graft degradation and tissue remodelling after 12 months of follow-up give rise to the question whether the valve can fully remodel towards a living valve in the high pressure circulation and what timeframe would be required. To this end, it is important to further investigate the cause of differences in cell colonisation and scaffold resorption observed between similar valves implanted in pulmonary position^13^ and aortic position (present study).

Possibly, the lack of pre-coating with fibrin, previously used in similar elastomeric valves implanted in pulmonary position^13^, could have contributed to decreased cell infiltration. Pre-coating of the scaffolds with fibrin, prior to implantation might promote cell infiltration and would be interesting to implement in future studies. In addition, the harsh hemodynamic environment of the aortic valve may limit cell infiltration. Many cells, including immune and tissue-producing cells, are known to be responsive to their mechanical environment and might, therefore interact differently on the same scaffold implanted in either low- or high-pressure circulation. This is underlined by results from a study that showed a reduced cell infiltration and migration rate when implanting decellularized xenograft patches (Cormatrix) in the aortic valve position in a clinical setting^37^. Our results indicate that long-term functionality of pliable elastomeric heart valves can be obtained without cell colonisation in the scaffolds. In fact, we observed the best functionality in valves that did not show any cellular infiltration nor scaffold degradation. Thus, a potentially interesting route to clinical application could be to change the scaffold material into a non-degradable material to form durable, non-living, thin and pliable synthetic heart valves. It is generally hypothesised that the use of non-degradable and non-living synthetic heart valves does not give a risk for leaflet thickening or retraction^39^. Properties and durability of synthetic non-degradable and non-living (e.g., polyurethanes and ultra-high molecular weight polyethylenes) materials for heart valve implantation have been optimised over the past years, including tear strength and creep resistance^40–42^. Typically, non-living synthetic heart valves will lack the potential of growth and remodelling. However, this non-degradable heart valve material can also be enclosed by the patient’s autologous cells, resulting in “hybrid valves’ that potentially have some repair and remodelling capacities^39,43^.

Lastly, we observed heterogeneity in results, specifically intervalve variability in terms of divergent remodelling pathways. Four valves showed holes in one of more leaflets (#6.1, #9.1, #12.1, #12.2), resulting in poor valve functionality and increased regurgitation as measured by ultrasound. Heterogeneity in remodelling of in situ tissue engineered heart valves has previously been reported in pulmonary pre-clinical studies including PC-BU based pulmonary valves^16^. With respect to the heterogenic results of the present study, it is essential to understand the cause of this variability as it has important safety implications when translating the technology to the clinic.

In conclusion, we here report on in situ tissue engineered heart valve prostheses implanted in the aortic position of an ovine model with prolonged follow-up of up to 12 months. Although grafts were designed to attract host cells to stimulate endogenous tissue formation, limited cell infiltration was observed, correlating to limited progression in tissue remodelling and scaffold resorption. Importantly, these valves may meet several hallmarks for sustainable heart valve prostheses, as all valves remained free from endocarditis, thrombotic complications and macroscopic calcifications throughout the 12 months follow-up. However, we have observed variability of the outcomes, including valves that showed minor microscopic calcification, leaflet thickening and retraction and valves that had holes in a leaflet. With this study we give insights in the tissue growth and remodelling on biodegradable elastomeric heart valves implanted in the high-pressure circulation, and conclude that 1-to-1 translation of results obtained by pulmonary valve implants cannot be imposed to aortic valve prostheses. As such, the present study has given new perspective on pliable elastomeric (bioresorbable) grafts as aortic valve replacement and can be considered as a promising starting point for improved sustainable aortic valve replacements.

## Methods

### Valve manufacturing

We fabricated aortic valve prostheses from electrospun scaffolds made of bis-urea-modified polycarbonate (PC-BU; fibre ø 3.6 ± 0.27 µm). In brief, PC-BU (SyMO-Chem BV, The Netherlands) was dissolved in a mix of solvents: chloroform (372978, Sigma-Aldrich, Saint Louis, USA) and hexafluoroisopropyl alcohol (Fluorochem Ltd., Glossop, UK) at a ratio 85:15 (v/v), to create 24% (w/v) homogenous solution. The obtained polymer solution was transferred to a syringe, which was placed in a syringe pump, that provided the feeding via Teflon tubing to the electrospinning apparatus (IME Medical Electrospinning, Waalre, the Netherlands). The electrospinning apparatus was equipped with a climate-controlled chamber, set up at 23 °C and 50% relative humidity. The polymer solution was electrospun on a mandrel target (ø = 20 mm) rotating at 195 RPM with a constant flow rate of 40 µl/min and a horizontal nozzle speed movement of 70 mm/s. To stabilise the fibre jet formation, a gas shield module was implemented (chloroform at 30 ml/min). The voltage applied at the nozzle was 17 kV and a linear gradient from 0 to −2 kV at the target side with time. Polyether ether ketone (PEEK) crown-shaped supporting stents/rings (external ø = 19 mm) were manufactured at the Equipment & Prototype Centre, Eindhoven University of Technology (Eindhoven, The Netherlands). Subsequently, the electrospun tubular conduits were sutured (6-0 Prolene C1, Ethicon, Somerville, US) onto the PEEK supporting ring to create a tri-leaflet valvular shape, as previously described in detail^13^, resulting in tri-leaflet heart valve prostheses. We have used PEEK rings with 19 mm outer diameter for aortic valve replacement. Prior to implantation, the valves were sterilised by ethylene oxide (Synergy health, Venlo, the Netherlands).

### Animals and surgical procedure

We surgically implanted the TE aortic valve prostheses in 20 female Swifter sheep in orthotopic position on the arrested heart after removing the native aortic valve. Approval for the animal studies was obtained by the Amsterdam University Medical Centers Animal Care Ethics Committee (AVD1180020197705) and are in agreement with the current Dutch law on animal experiments (WOD). We have complied with all relevant ethical regulations for animal testing. The animals came from a local farm and were bred for education purposes. The animals were quarantined for at least 14 days prior to surgery. We conducted a detailed animal welfare assessment once a week, during which all animals were thoroughly checked for any clinical symptoms or discomfort. We housed all animals in groups in an indoor shelter with ad libitum access to food and water. In total 20 sheep underwent chronic implantation with our aortic valve substitute (mean age 1.97 ± 0.23year, mean weight 69.4 kg ±6.6 kg). The surgical procedures were performed in an experienced animal laboratory by an experienced cardiac surgeon (J.K). We applied a buprenorphine patch (5 mcg/h patch; BuTrans, Mundipharma, Cambridge, UK) on the ventral side of the proximal part of the tail, one day prior to the aortic valve surgery. On the day of surgery, we administered ketamine hydrochlorine (10 mg/kg IM, narketan 100 mg/ml, Vétoquinol, Paris, France) and midazolam (0.4 mg/kg IM, midazolam Actavis 5 mg/ml, Actavis, New Jersey, US) as pre-anaesthetic medication. Prior to incision, we administered a single dose of amoxicillin/clavulanic acid (20 mg/kg IV, amoxicillin/clavulanic acid 500 mg/50 mg, Sandoz, Holzkirchen, Germany), for antibiotic therapy. We used propofol to induce anaesthesia (2–4 mg/kg IV; propofol 20 mg/ml, Fresenius Kabi, Bad Homburg, Germany) and to maintain anaesthesia (20 mg/kg/h IV) during surgery. We used sufentanil (5 mcg/kg/h IV, sufentanil-Hameln 50 mcg/ml, Hameln, Gloucester, UK) as pain relief during surgery. We added a single dose of amiodarone hydrochlorine (300 mg IV, cordarone 50 mg/ml, Sanofi, Paris, France) in the saline infusion bag before starting cardiopulmonary bypass (CPB). During surgery, all animals were monitored by means of ECG, arterial blood pressure and capnography. Animals were placed in the right lateral position and the left thorax and neck were sterilised. They were placed on CPB after heparinization (15000–20000 IU IV, heparine 5000 IU/ml, LEO, Ballerup, Denmark). We used CPB flow rates between 3.5–4 L/min. An activated clotting time >400 s was maintained during CPB and arterial blood gas analyses were performed. For arterial access, a 16 Fr arterial cannula (Edwards Lifescienes, Irvine, US) was inserted in the left carotid artery. A 24 Fr venous cannula (Edwards Lifesciences, Irvine, US) was inserted in the left jugular vein. We used the Seldinger technique for both canulation sites. A left-sided anterolateral thoracotomy was performed in the third or fourth intercostal space. The pericardium was opened and the pulmonary artery was dissected free from the aorta. A 13 Fr cannula (Medtronic, Minneapolis, US) was placed in the left ventricle via the left atrial appendage for left ventricular venting. The animal was cooled down to 32 °C. The ascending aorta was clamped just proximal to the junction of the brachiocephalic trunk. A needle was inserted into the aorta proximal to the cross clamp and one litre of cardioplegic solution was administered into the ascending aorta. We used the commercially available formulation of St. Thomas’ Hospital Cardioplegic Solution no. 1 as basis to which we added mannitol (80 ml; mannitol 10%, Baxter, Utrecht, the Netherlands). We administered the cardioplegia solution at a flow-rate of 250 ml/min. Cold saline was poured directly in the pericardial space. A subtotal transverse aortotomy was made distally from the sinotubular junction after which the cusps of the native aortic valve were excised. The heart valve prosthesis was implanted using nonpledgeted single interrupted sutures (2-0 Ti-Cron, Covidien, Dublin, Ireland). The aorta was closed in a continuous fashion (5-0 Prolene C1, Ethicon, Somerville, US) and carefully de-aired. The aorta clamp was removed. If needed, the aorta was re-clamped and/or the heart was defibrillated (20 J) in order to convert to a stable sinus rhythm^44^. The animal was rewarmed. The left venting cannula was removed and the incision in the left auricular appendage was closed with a running suture (5-0 Prolene C1, Ethicon, Somerville, US). After weaning from extracorporeal circulation, an epicardial echocardiography using a transesophageal echo probe on a Philips iE33 ultrasound machine was made to assess valve function. Protamine was administered intravenously before removal of the cannulas. A chest tube was placed in the left thoracic cavity. The wound was closed in layers. The chest tube was removed shortly before extubation. During the first three days after surgery flunixinemeglumine (0.02 ml/kg IM, niglumine, 50 mg/ml, Dopharma, Raamsdonksveer, Netherlands) was administered. All animals received furosemid (20 mg IM, Centrafarm, 20 mg/2 ml, furosemide, Etten Leur, Netherlands), neomycine/procaine benzylpenicilline (1 ml/20 kg IM, neopen, 100 mg/200.000 IE/ml, Intervet, Boxmeer, Netherlands) and nadroparin (3800 IE IM, Fraxiparine, 9.500 IE/ml, Aspen, Durban, South Africa) during the first 7 days after surgery. During follow-up, the animals received carbasalate calcium (Ascal 80 mg orally; Sandoz, Basel, Switzerland) for 3 months.

### Functional valve evaluation

At the time of explantation, we conducted transthoracic echocardiography using a Philips iE33 ultrasound machine to assess in vivo valve performance under full anaesthesia. After completing ultrasound measurements, we performed a sternotomy and invasively measured the blood pressures in the left ventricle and the ascending aorta. We exsanguinated the animal and evaluated the transplanted heart valve macroscopically. Echo recordings were qualitatively evaluated in a blinded fashion by two experienced clinicians (M.V. and L.G.) on aortic insufficiency (grade mild/moderate/severe) and on aortic stenosis (mild/moderate/severe). Aortic insufficiency was scored on regurgitant jet area, jet width and pressure half time. Aortic stenosis was scored by manually tracing continuous wave (CW) Doppler derived flow velocity curves, by which the peak- and mean transvalvular flow velocities were measured. Using the simplified Bernoulli equation (Δp = 4V2) transvalvular peak- and mean pressure gradients were derived. In addition, pulsed wave (PW) derived flow velocity in the left ventricular outflow tract was measured, allowing for the estimation of the aortic valve area (AVA) using the continuity equation.

### Explant evaluation

During valve explantation we assessed the left and right ventricle on being hypertrophic or dilated. We checked for presence of effusion in thorax or abdomen or presence of thrombi in heart or vessels. The bioresorbable synthetic aortic heart valves were macroscopically evaluated for signs valve deterioration (such as degradation, holes in the leaflets or leaflet retraction), additional tissue growth and pliability. A suture for orientation was placed between the right coronary and left coronary leaflet, and the valve was carefully resected from the PEEK-reinforcement ring. The valve was cut in longitudinal direction between the commissures of the left and right cusp to expose the leaflets, and photos were taken for gross morphology assessment. The valve was carefully resected from the PEEK ring and transsected for further analysis according to a previous defined cutting scheme (Supplementary Fig. 8).

### Histological analysis

Sections of each valve leaflet, and samples of the liver, lung, kidney and spleen were fixated in 3.7% formalin for 24 h, washed in phosphate buffered saline (PBS) and embedded in paraffin. Longitudinal sections of 5 µm were cut, deparaffinized in xylene and rehydrated in a graded ethanol series. Afterwards, valve sections were stained for hematoxylin and eosin (H&E; HT1079 and HT110116, Sigma-Aldrich, Saint Louis, US), Movat Pentachrome (American MasterTech, Lido, US) for gross tissue morphology, Alizarin Red (Abcam, Cambridge, UK) for calcific deposits, Elastica van Gieson (EvG;, VWR, Radnor, US) for elastic fibres and Picrosirius Red (VWR, Radnor, US) for collagen content. All stained sections were digitised using a Philips Ultra Fast Scanner (Philips Digital Pathology Solutions, Best, the Netherlands) and high-resolution images were saved as Tiff (resolution: 4 pixels/μm). Qualitative histological analyses were conducted by four reviewers (A.V., B.K., N.V. & E.L.) and were supervised by a clinical pathologist (H.N.). During the semi-qualitative histological analysis, the reviewers were blinded for the follow-up times of the animals at all times. We structurally analysed a hinge, belly and tip regions of all valve leaflets. Sections of the liver, lung, kidney and spleen were stained for hematoxylin and eosin for each animal, except for the spleen of animal #3.2 that could not be retrieved. All sections were qualitatively assessed for pathological changes and vascular thrombi.

The protocol for the cell counting method was as follows: A magnification of 1x was used to obtain a general overview and to determine the locations of the hinge, belly and tip regions of each valve leaflet. We analysed one histological slice for each valve leaflet. For each histological slice, we selected squares of 0.4 mm2 at the tip region of the valve, meaning the most distal parts of each valve leaflets. Similarly, we selected the hinge region at the most proximal part of the valve leaflet, and the belly region at the lowest point of the curvature of the valve leaflet. First, the regions of interest were defined by reviewers N.V. and E.L independently. Afterwards, the location of all regions of interest was checked by reviewers A.V. and B.K. until consensus was reached.

If the cell count in each region of interest was less than 10, we counted the cells manually. If the cell count was greater than 10, we used ImageJ software to count the cells. We used the same ImageJ protocol for all regions of interests.

Please find the ImageJ protocol for cell count below:

1. Image -> Colour -> split channels
2. Process -> Image calculator -> create a new window using the red and green images with the function ‘average’
3. Process -> Subtract background -> Rolling ball radius: 7.5 pixels -> check the box with ‘Light background’
4. Process -> Filters -> Gaussian blur -> Sigma radius=1.00
5. Process -> Find maxima -> check the box with ‘Light background’ and ‘Preview point selection’ -> Prominence > 30.00
6. Amount of maxima (=cell nuclei) is seen on the bottom of the window

After determining the cell count, the surface area of each region of interest was calculated. Using Digipath software (Philips), the area of the histological sample was encircled using the “freeform” tool, after which the surface was displayed. The number of cells was divided by the total tissue area, and means and standard deviations (±) were calculated. Initial cell count and surface area measurement was performed by reviewers N.V. and E.L. independently from each other. Their findings were compared, and if no consensus was reached, cell count and or surface area measurement was repeated by a third reviewer (A.V.) until consensus was found.

In order to quantify the area of the valve leaflet covered by collagen, we have identified square shaped regions of interests (0.9 mm2) in hinge, belly and tip regions in one slide stained with Picrosirius Red for each valve leaflet. The total tissue surface within the region of interest, as well as the total surface of collagen were both calculated using Fiji software. The collagen area was then divided by the total tissue area, and means and standard deviations (±) were calculated. Analysis to quantify the calcification in the leaflets, was performed based on a previously published quantification system by Gomez-Stallons et al.^25^. We have analysed the hinge, belly and tip regions in one slice for each valve leaflet stained with Alizarin Red staining. Each leaflet sample was assigned a qualitative calcification grade value per leaflet region. The method was modified as follows:

-Grade 0 No calcification
-Grade 1 Mildly calcified, small isolated lesions
-Grade 2 Moderately calcified, multiple larger lesions
-Grade 3 Severely calcified, extensive calcification throughout.

We calculated an average calcification grade for each valve leaflet. Valves were categorised as Non-calcified ( < 0.10), Mild (score >0.10 and <0.75) or Moderate (score > 0.75), based on the average calcification grade.

### Biochemical analysis

Explanted valves were quantified for DNA, hydroxyproline (HYP) as measure of collagen content, and glycosaminoglycans (GAG). Briefly, valves were lyophilized and weighted to determine dry weight and afterwards snap frozen and disrupted by a dismembrator (Sartorius, Göttingen, Germany). Samples were dissolved in 500 µl digestion buffer containing 100 nM phosphate buffer, 5 mM L-cystein, 5 mM ethylenediaminetetraacetic acid, papain (Sigma) and ultrapure water (MiliQ, Millipore MiliQ systems) and digested overnight at 60 °C. GAG and HYP assays were performed as described elsewhere^45,46^. DNA content was determined using high sensitivity dsDNA measurement of Qubit assay kit (ThermoFisher Scientific), following the protocol of the manufacturer. All values were normalised to the samples dry weight.

### Scanning electron microscopy (SEM) and gel permeation chromatography (GPC)

To determine graft resorption two methods were used, i.e. SEM and GPC. GPC was performed to gain more insight in the possible degradation and resorption mechanism of the synthetic material. It is a quantitative method analysing the molecular weight of the polymer/polymer fragments as well as the polymer weight distribution. As SEM can only visualise the top layer of the material, GPC is a good method to gain insight of the degradation of the material throughout the full depth of the valve. First, neo-tissue of the explants was removed by 4.6% (v/v) sodium hypochlorite (Clorox) incubation for 15 min at room temperature. Afterwards samples were washed twice in MiliQ and dehydrated in a graded ethanol series for scanning electron microscopic analysis. The samples were subsequently dried using a critical point dryer (CPD300, Leica, Wetzlar, Germany) and samples were visualised by SEM (Quanta 600 F, GEI, Eindhoven, the Netherlands). For GPC analysis washed samples were allowed to dry and, along with two control samples consisting of implant material, dissolved in dimethylformamide with 0.25% (v/v) in water and 0.1% (w/w) lithium bromide with an approximate concentration of 1 mg sample per ml. After sample filtration with an 0.2 µm filter, number averaged molecular weight (Mn) and weight-averaged molecular weight (Mw) were determined at 50 °C by a Varian/Polymer Laboratories PL-GPC 50 using a Shodex GPC KD-804 column.

### Biaxial tensile testing

Mechanical behaviour of the explanted valve was determined by biaxial tensile testing of squared pieces (5 × 5 mm²) cut from the tip/belly region of the valve. Similarly, mechanical behaviour of electrospun material before implantation was tested, for which pieces were cut from the leftover implant material. Sample thicknesses were measured by digital microscopy (VHX-500FE, Keyence, Mechelen, Belgium), and were averaged from all sides of each sample. Subsequently, samples were placed in the tensile tester (BioTester 5000, CellScale, Canada, 1,5 N load cell, using LabJoy software V8.01) and stretched equibiaxially in the radial and circumferential direction. Strain was increased stepwise by 5% to a maximum strain of 40%, each step consisting of 9 pre-stretching cycles and 1 measurement cycle. Stress-strain curves and tangent moduli presented were determined at 35% strain.

### Statistics and reproducibility

Means and standard deviations were calculated for cell count per mm^2^, DNA, HYP and GAG measurements, collagen ratio, GPC measurement and mechanical testing. All sheep experiments were conducted once. We did not reproduce the results.

### Reporting summary

Further information on research design is available in the Nature Portfolio Reporting Summary linked to this article.

### Supplementary information

Supplementary Information

Description of Additional Supplementary Files

Supplementary Movie 1

Supplementary Movie 2

Supplementary Movie 3

Supplementary Data 1

Reporting Summary

## Data Availability

Numerical source data for graphs and charts are available in the Supplementary Data 1. Access to materials for reproducing all other data (histological slides, scaffolds, etc) can be obtained from the corresponding authors upon reasonable request.
