# Supplementary material for: Evaluation of pliable bioresorbable, elastomeric aortic valve prostheses in sheep during 12 months post implantation
Source: Commun Biol. 2023 Nov 14;6:1166. doi: 10.1038/s42003-023-05533-3 (PMC10646052; doi:10.1038/s42003-023-05533-3)
Supplement: Supplementary file 1 — Supplementary Information [file 42003_2023_5533_MOESM1_ESM.pdf]

SUPPLEMENTARY MATERIALS AND METHODS

Evaluation of pliable bioresorbable, elastomeric aortic valve prostheses in sheep during 12 months post implantation.

Authors: Annemijn Vis[1]†, Bente J. de Kort[2,3]†, Wojciech Szymczyk[2,3], Jan Willem van Rijswijk[2,3], Sylvia Dekker[2,3], Rob Driessen[2,3], Niels Wijkstra[4], Paul F. Gründeman[5], Hans W.M. Niessen[6], Henk M. Janssen[7], Serge H.M. Söntjens[7], Patricia Y. W. Dankers[2,3], Anthal I.P.M. Smits[2,3], Carlijn V.C. Bouten[2,3]‡*, Jolanda Kluin[1,8]‡*

Affiliations:

[1]Department of Cardiothoracic Surgery, Amsterdam University Medical Centers location University of Amsterdam; Amsterdam, the Netherlands

[2]Department of Biomedical Engineering, Eindhoven University of Technology; Eindhoven, the Netherlands

[3]Institute for Complex Molecular Systems (ICMS), Eindhoven University of Technology; Eindhoven, The Netherlands

[4]Department of Cardiology, Amsterdam University Medical Centers; Amsterdam, the Netherlands

[5]Department of Cardiothoracic Surgery, University Medical Center Utrecht; Utrecht, The Netherlands

[6]Department of Pathology, Amsterdam University Medical Centers, ACS; Amsterdam, the Netherlands

[7]SyMO-Chem BV; Eindhoven, The Netherlands

[8]Department of Cardiothoracic Surgery, Thorax Center, Erasmus MC Rotterdam, the Netherlands

*Corresponding authors. Email: j.kluin@erasmusmc.nl; c.v.c.bouten@tue.nl.

Author notes:
† These authors contributed equally to this work
‡ These authors jointly supervised this work

20 sheep underwent heart valve implantation

5 surgical deaths related to the procedure and not to the valve itself

15 sheep survived surgery, and were randomized for follow up time:

1 month follow up, n=2
3 months follow up, n=3
6 months follow up, n=3
9 months follow up, n=3
12 months follow up, n=4

One sheep appointed to the 9 months follow up group, died unexpectedly after 6 months. Reason of death is unclear.

This sheep was analyzed as being part of the 6 months follow up group.

15 sheep are included in the analysis, with the following follow up times:

1 month follow up, n=2
3 months follow up, n=3
6 months follow up, **n=4**
9 months follow up, **n=2**
12 months follow up, n=4

Supplementary Figure 1. Flow chart of included and excluded animals during follow-up.

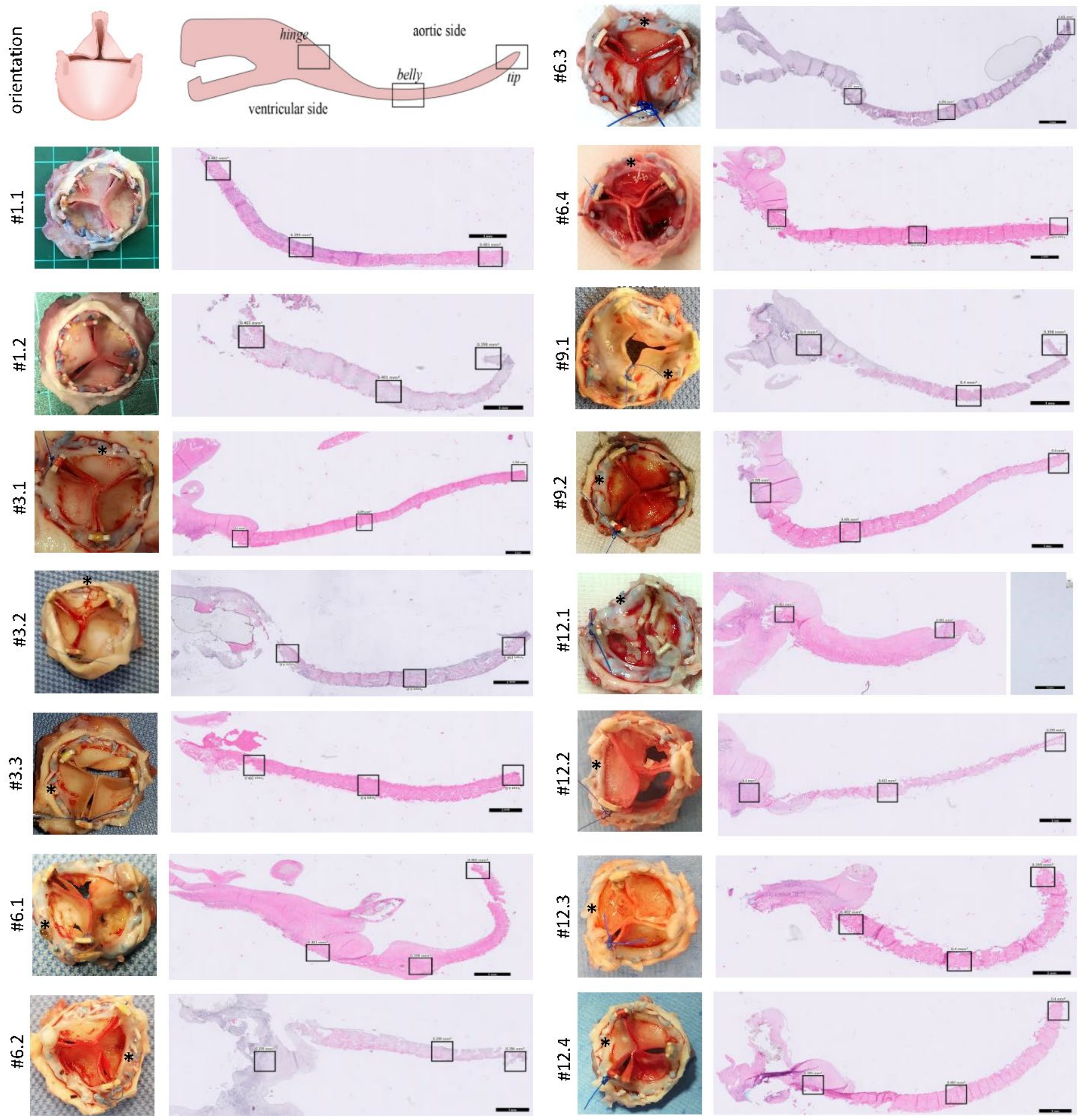

Supplementary Figure 2. Histological slices of all valves, stained with H&E. For each heart valve, a photograph of the valve after explant is depicted (left) accompanied by a longitudinal histological slice of one representative valve leaflet (right). The histological slices are stained with H&E staining. Hinge regions are depicted on the left side of the histological image and tip is depicted on the right side. Boxes indicate hinge, belly and tip regions of te leaflets. * indicates the leaflet of which the histological slide is shown. Scalebar 1mm.

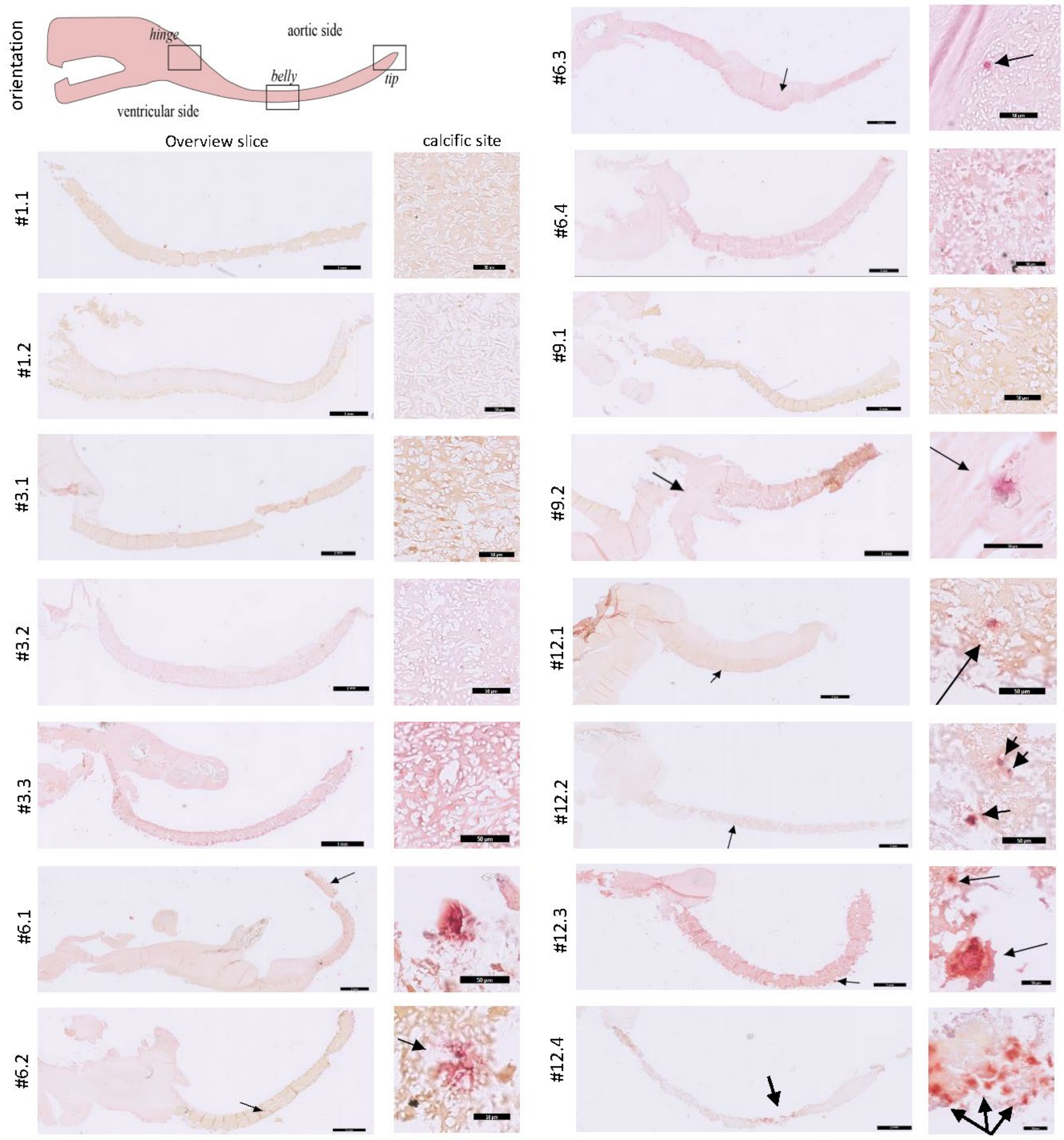

Supplementary Figure 3. Histological assessment of calcification in all valves. This figure shows one histological slide for each explanted heart valve, stained with Alizarin Red staining. Calcific sites turn red with Alizarin Red staining. The image of the left is an overview slide (scalebar 1mm), the image of the right is a close-up of a calcific region (scalebar 50µm). The calcific region that is depicted in the close-up, is indicated with an arrow on the left image. When the overview slide does not have any arrows, no calcific sites were seen. Valves #6.1, #6.2, #12.3 and #12.4 are classified as mildly calcified.

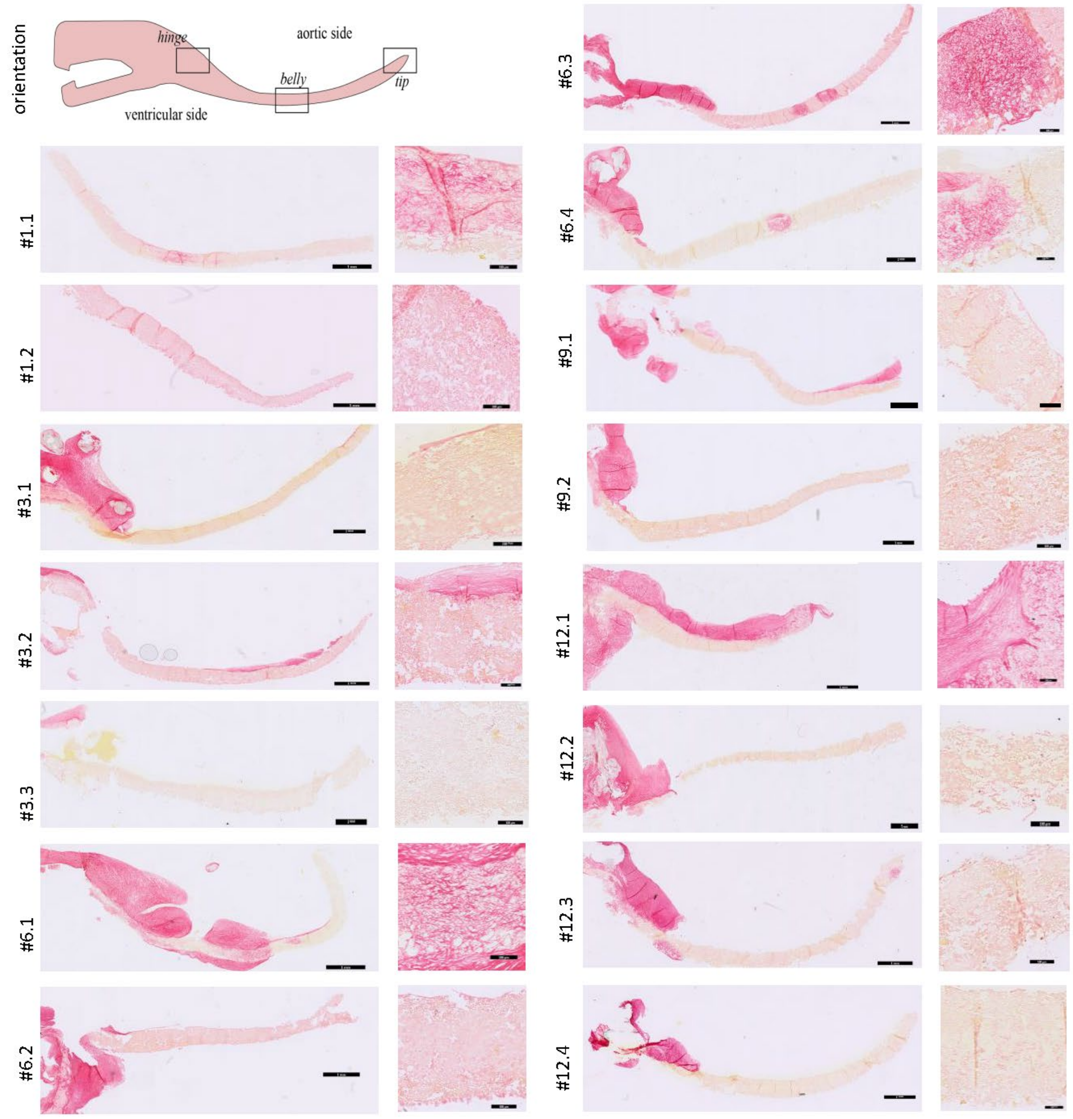

Supplementary Figure 4. Histological assessment of collagen deposition in all valves. This figure shows one histological slide for each explanted heart valve, stained with Picrosirius Red staining. The image of the left is an overview slide (scalebar 1mm), the image of the right is a close-up of the belly region (scalebar 100µm). Tissue that contains collagen turn red with this staining.

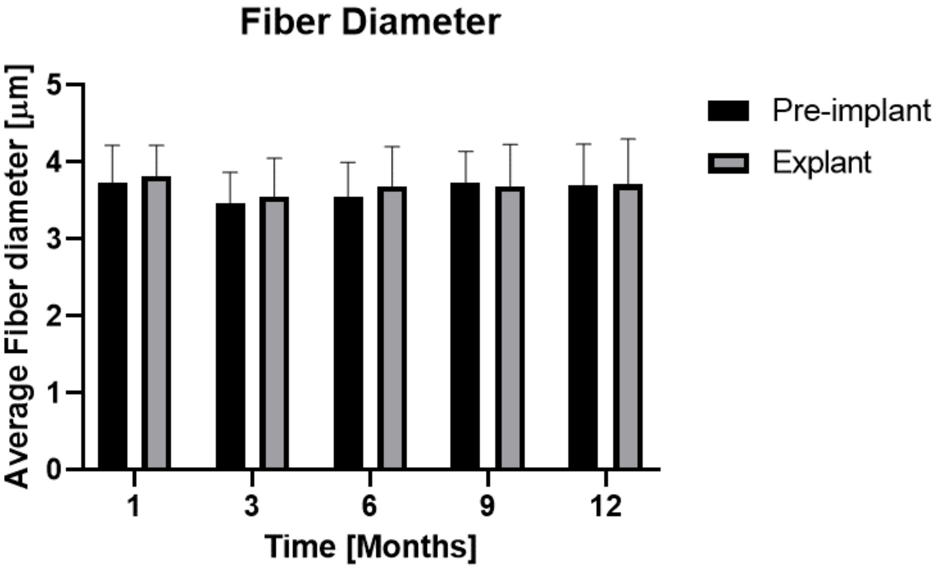

Supplementary Figure 5. Average fibre diameter of electrospun scaffolds before implantation and average fibre diameter of explanted valves. For all valves fibre diameters were measured in various locations on both sides (n=15-20 per location) using ImageJ. Diameters were averaged for explantation times; 1, 3, 6, 9 and 12 months. Mean values are reported in the bar chart. Error bars represent standard deviations. Total number of fibre diameter measurements used for calculating the mean values; 1 month pre implant n=120, 1 month explant n=240, 3 months pre implant n=180, 3 months explant n=360, 6 months pre implant n=240, 6 months explant n=480, 9 months pre implant n=90, 9 months explant n=240, 12 months pre implant n=240, 12 months explant n=300.

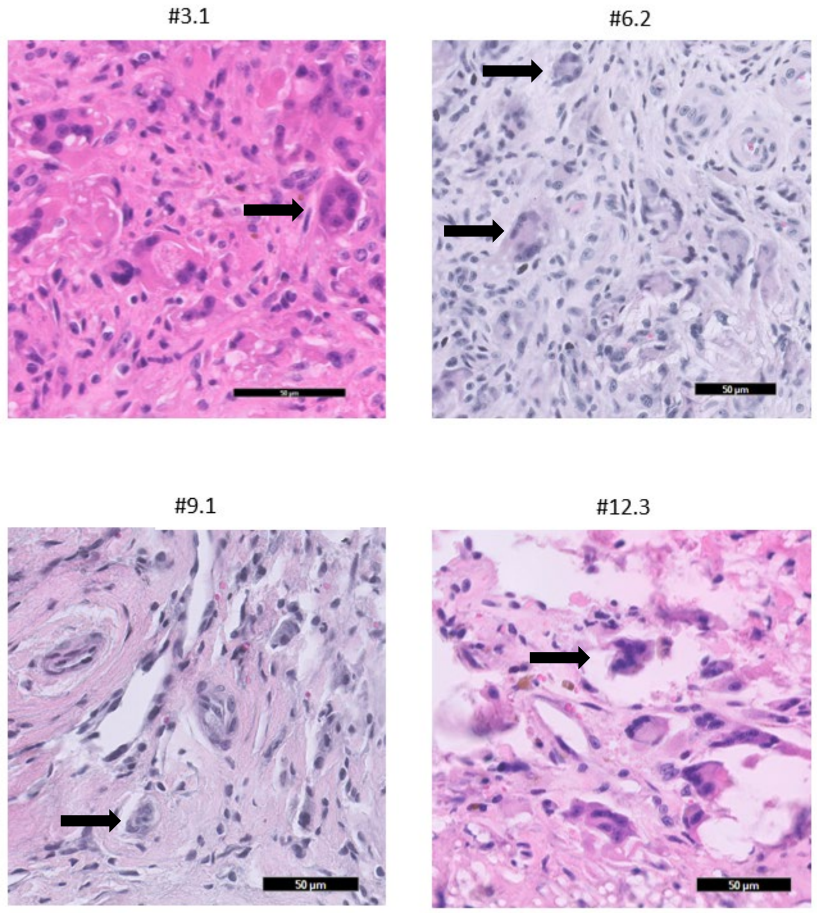

Supplementary Figure 6. Histological observation of giant cells. Representative regions of interest in hinge regions are shown for a representative valve at 3, 6, 9 and 12 months follow-up, stained with H&E. Giant cells (arrows) contribute to scaffold resorption. Scale bars 50µm.

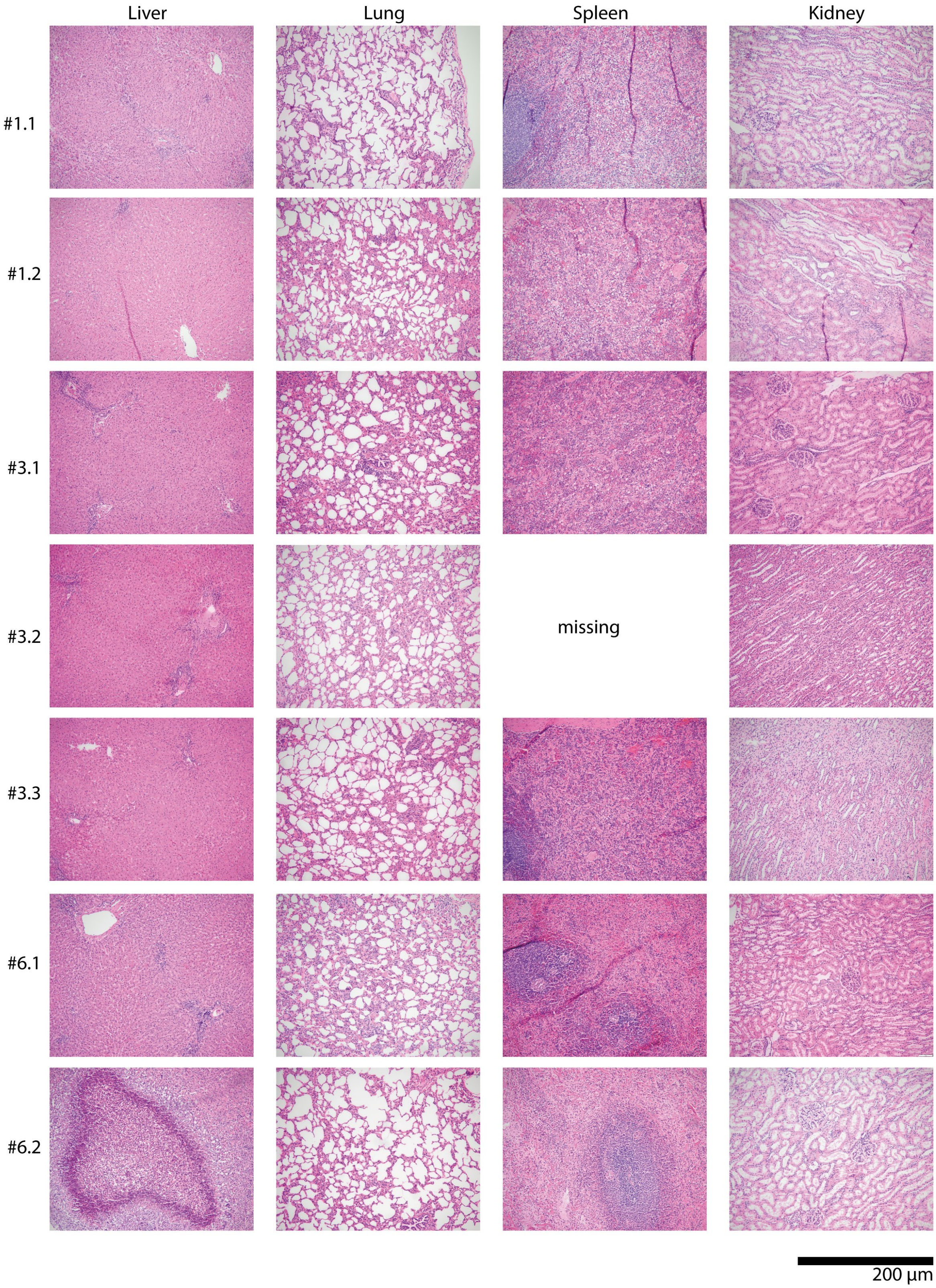
Liver
Lung
Spleen
Kidney
#1.1
#1.2
#3.1
#3.2
missing
#3.3
#6.1
#6.2
200 µm

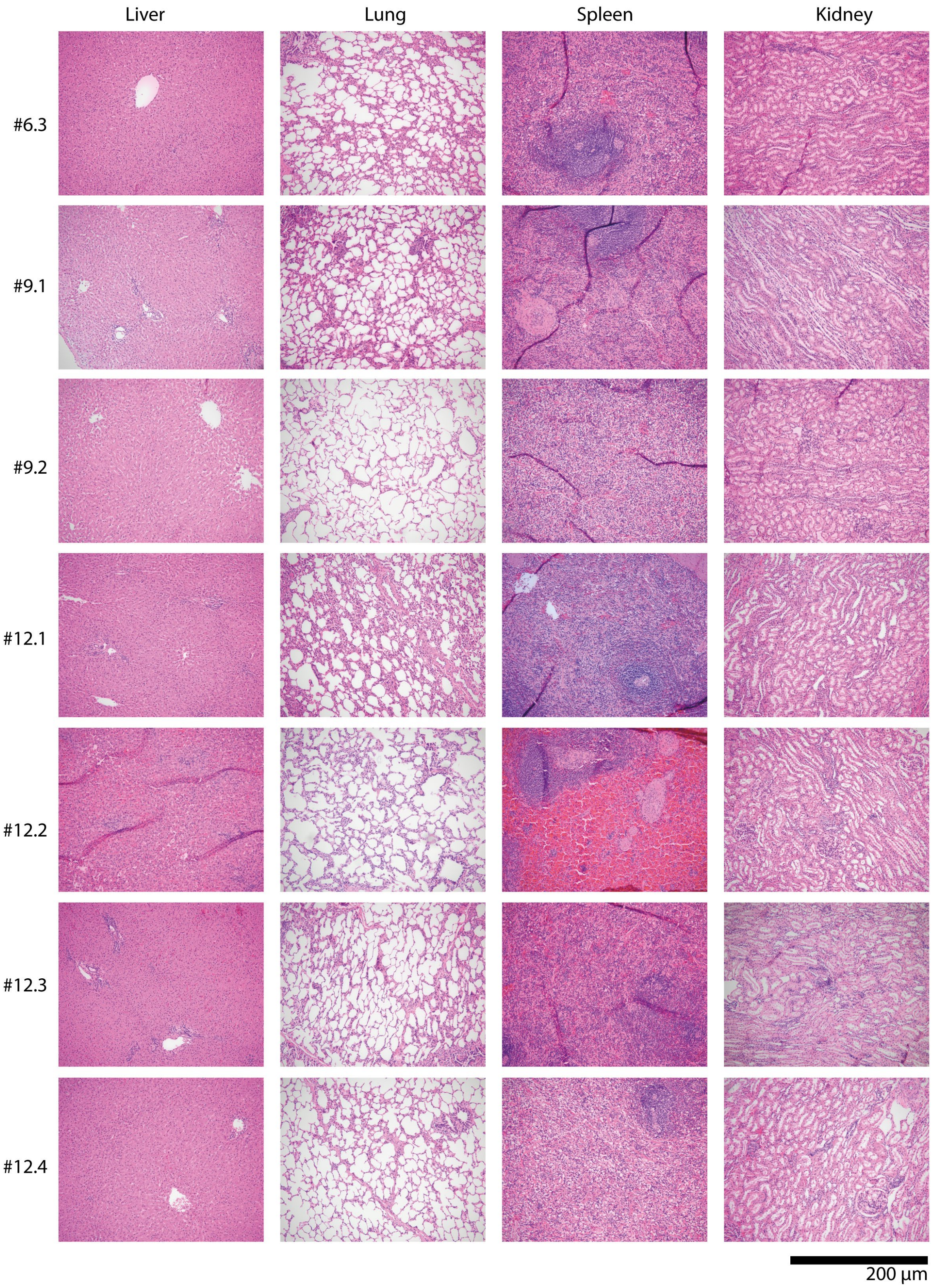

Supplementary Figure 7 (part I and II). Histological analysis of the liver, lung, spleen and kidney. All samples are stained with H&E. No recent or older thrombi were found in the examined organs. Also no major pathological changes of the organs were found, except for a non-specific acute inflammation of the liver but without foreign body granulomas (#6.2).

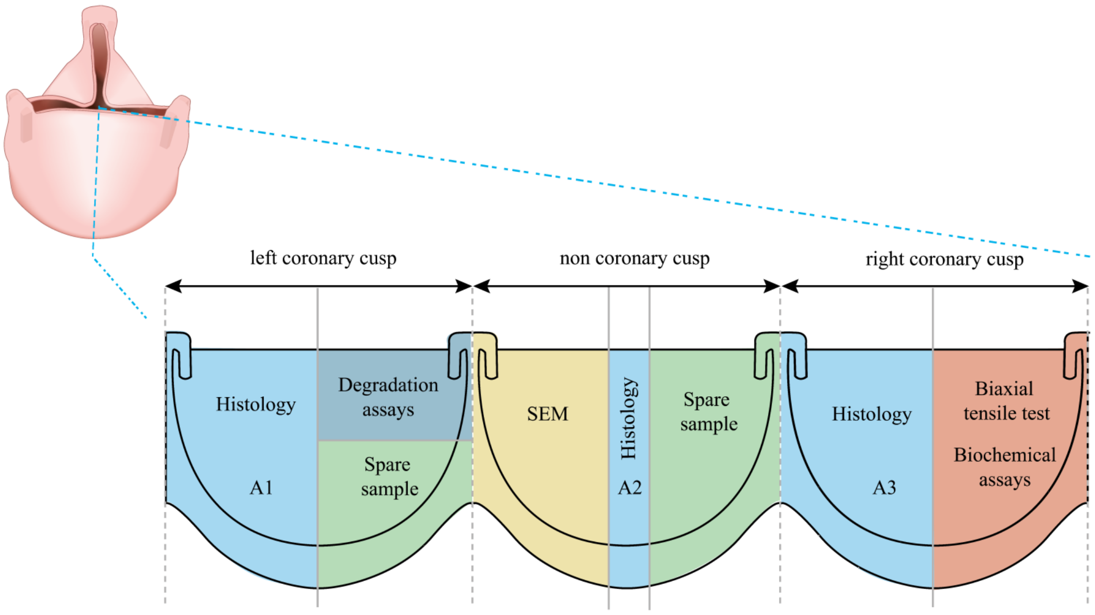

Supplementary Figure 8. Cutting scheme for valve analysis. SEM=Scanning Electron Microscopy. Green areas are spare samples and not yet analysed. For right coronary cusp 2 analyses were performed on same area, first pieces were used for biaxial tensile testing and subsequently whole area was used for biochemical assay.

Supplementary Table 1: In vivo echographic assessment of valve functionality.

Valve number	**#1.1**	**#1.2**	**#3.1**	**#3.2**	**#3.3**	**#6.1**	**#6.2**	**#6.3**	**#9.1**	**#9.2**	**#12.1**	**#12.2**	**#12.3**	**#12.4**
Heart Rate (BPM)	80	92	96	60	120	80	108	90	83	115	100	170	110	120
Systolic pressure (mmHg)	80	90	x	105	121	150	101	100	160	100	120	77	101	86
Diastolic pressure (mmhg)	62	60	x	80	101	45	80	80	100	80	75	51	80	65
Doppler velocity index	0,34	0,41	0,56	x	0,49	x	0,8	0,52	x	0,33	0,24	x	0,33	0,35
LVOT velocity; V1 (m/s)	>1.4	0,9	1,2	x	1	x	1,4	1,2	x	0,9	1	x	0,8	1,1
Peak Jet Velocity; Vmax (m/s)	2,8	2,4	2,1	x	2,1	2,9	2,2	2,3	4,3	2,7	4,6	>2	2,3	3
Peak Gradient (mmHg)	31	23	18	x	18	35	20	25	74	28	85	x	22	52
Mean Gradient (mmHg)	15	11	8	x	8	18	10	12	34	15	47	x	11	24
Valve Stenosis Grade	None	Mild	None	None	None	None	None	None	Moderate	None	Severe	None	None	Mild
Regurgitant flow	Yes	Yes	No	Yes	Yes	Yes	Yes	Yes	Yes	No	Yes	Yes	Yes	Yes
Number of jets (estimation)	3	>2	NA	x	2	x	x	x	2	NA	x	x	x	>2
eccentric/central jet?	x	Eccentric	x	x	x	x	x	Central	Eccentric	x	Eccentric	x	x	x
valvular/paravalvular jet?	Paravalvular	Paravalvular	x	Valvular	Valvular	Valvular	Valvular	Valvular	x	x	Paravalvular	x	x	Valvular
Jet Width	x	x	x	x	x	large jet	x	x	x	x	large	large	x	x
Pressure halftime	x	x	x	x	x	Short	x	Long	x	x	Short	x	x	x
Other findings/comments	1 paravalvular jet	1 excentric jet, 1 paravalvular jet	x	x	x	AR Vmax 3 m/s (pressure equilibration)	x	x	1 excentric jet, 1 central jet.	x	x	AR Vmax 3 m/s (pressure equilibration)	very mild	x
Regurtitation Grade	Moderate Non-structural	Mild	None	Mild	Mild	Severe	Mild	Mild	Moderate	None	Severe	Severe	Mild	Mild
Valve morphology	x	Turbulent flow ao asc, good valve opening (short acceleration time)	normal valve opening, no turbulent flow	normal valve opening	normal valve opening	two valve scallops move excessively. High gradient due to severe regurgitation	doming one aortic valve cusp	x	Turbulent flow, moderate stenosis of aortic valve	x	1 valve scallop moves excessively (flail)	turbulent flow	Norma valve opening	valve scallops move excessively
LV function	Mild abnormal	Normal	Normal	Normal	Normal	Normal	Normal	Normal	Normal	Mild abnormal	Mild abnormal	Severe abnormal	Normal	Normal
LV dilatation	No	No	No	No	No	Yes	No	No	No	No	Yes	Yes	No	No
LV hypertrophy	No	No	Yes	No	No	No	Yes	Yes	Yes	Yes	No	No	Yes	Yes
RV function	Normal	Normal	Mild abnormal	Normal	Normal	x	Normal	Normal	Unclear	Normal	Mild abnormal	Severe abnormal	Mild abnormal	Normal
Pericardial Effusion	No	No	No	No	No	No	No	Yes	Yes	No	No	No	Yes	No
Other findings	wall motion abnormalities apical	x	x	x	x	mildly dilatated left ventricle	Dilatated ascending aorta	hypokinesia septal	Dilatated ascending aorta	x	x	pleura effusion	x	x

Supplementary Table 2: In vivo invasive assessment of valve functionality.

Valve number	**#1.1**	**#1.2**	**#3.1**	**#3.2**	**#3.3**	**#6.1**	**#6.2**	**#6.3**	**#9.1**	**#9.2**	**#12.1**	**#12.2**	**#12.3**	**#12.4**
P LVOT systolic, mmHg	X	X	X	137	102	66	91	100	76	86	86	29	104	35
P LVOT diastolic, mmHg	X	X	X	124	90	44	74	87	52	76	58	23	78	25
P LV systolic, mmHg	X	X	X	170	130	100	118	133	122	112	133	42	123	36
P LV diastolic, mmHg	X	X	X	0	0	0	6	3	0	4	4	25	-3	5
Transvalvular ΔP peak-to-peak gradient, mmHg	X	x	X	33	28	34	27	33	46	26	47	13	19	1
Comment												Hemodynamic unstable		Hemodynamic unstable
Comment												Excluded from analysis		Excluded from analysis
