# Supplementary material for: Evaluation of pliable bioresorbable, elastomeric aortic valve prostheses in sheep during 12 months post implantation
Source: Commun Biol. 2023 Nov 14;6:1166. doi: 10.1038/s42003-023-05533-3 (PMC10646052; doi:10.1038/s42003-023-05533-3)
Supplement: Supplementary file 2 — Description of Additional Supplementary Files [file 42003_2023_5533_MOESM2_ESM.pdf]

Description of Additional Supplementary Files

File name: Supplementary Movie 1
Description: 1Echographic images of a representative valve (#1.2) shortly after implantation. Good opening and closing behaviour of all three leaflets is shown. Doppler measurements show that there is no regurgitant flow. Leaflet coaptation length is 0.49cm. Dist = distance.

File name: Supplementary Movie 2
Description: Echographic images of a representative valve (#6.2) after 6 months follow-up. Normal left ventricular function. Good coaptation of the valve leaflets. Mild regurgitant flow is observed. No valve stenosis.

File name: Supplementary Movie 3
Description: Echographic images of a representative valve (#12.3) after 12 months follow-up. Normal left ventricular function. Normal valve opening. Very mild regurgitant flow is observed. No valve stenosis.

File name: Supplementary Data 1
Description: The source data behind the graphs in the paper.
