# Supplementary material for: Evaluation of pliable bioresorbable, elastomeric aortic valve prostheses in sheep during 12 months post implantation
Source: Commun Biol. 2023 Nov 14;6:1166. doi: 10.1038/s42003-023-05533-3 (PMC10646052; doi:10.1038/s42003-023-05533-3)
Supplement: Supplementary file 7 — Reporting Summary [file 42003_2023_5533_MOESM7_ESM.pdf]

nature portfolio

Corresponding author(s): J. Kluin, j.kluin@erasmusmc.nl; C.V.C. Bouten, c.v.c.bouten@tue.nl

Last updated by author(s): Oct 9, 2023

Reporting Summary

Nature Portfolio wishes to improve the reproducibility of the work that we publish. This form provides structure for consistency and transparency in reporting. For further information on Nature Portfolio policies, see our Editorial Policies and the Editorial Policy Checklist.

Statistics

For all statistical analyses, confirm that the following items are present in the figure legend, table legend, main text, or Methods section.

n/a	Confirmed	
☒	☐	The exact sample size (*n*) for each experimental group/condition, given as a discrete number and unit of measurement
☒	☐	A statement on whether measurements were taken from distinct samples or whether the same sample was measured repeatedly
☒	☐	The statistical test(s) used AND whether they are one- or two-sided<br>*Only common tests should be described solely by name; describe more complex techniques in the Methods section.*
☒	☐	A description of all covariates tested
☒	☐	A description of any assumptions or corrections, such as tests of normality and adjustment for multiple comparisons
☒	☐	A full description of the statistical parameters including central tendency (e.g. means) or other basic estimates (e.g. regression coefficient) AND variation (e.g. standard deviation) or associated estimates of uncertainty (e.g. confidence intervals)
☒	☐	For null hypothesis testing, the test statistic (e.g. *F*, *t*, *r*) with confidence intervals, effect sizes, degrees of freedom and *P* value noted<br>*Give P values as exact values whenever suitable.*
☒	☐	For Bayesian analysis, information on the choice of priors and Markov chain Monte Carlo settings
☒	☐	For hierarchical and complex designs, identification of the appropriate level for tests and full reporting of outcomes
☒	☐	Estimates of effect sizes (e.g. Cohen's *d*, Pearson's *r*), indicating how they were calculated

Our web collection on statistics for biologists contains articles on many of the points above.

Software and code

Policy information about availability of computer code

Data collection	no computer code was used
Data analysis	no computer code was used

For manuscripts utilizing custom algorithms or software that are central to the research but not yet described in published literature, software must be made available to editors and reviewers. We strongly encourage code deposition in a community repository (e.g. GitHub). See the Nature Portfolio guidelines for submitting code & software for further information.

Data

Policy information about availability of data

All manuscripts must include a data availability statement. This statement should provide the following information, where applicable:

- Accession codes, unique identifiers, or web links for publicly available datasets
- A description of any restrictions on data availability
- For clinical datasets or third party data, please ensure that the statement adheres to our policy

All data are available in the main text or the supplementary materials. Access to materials for reproducing the data (histological slides, scaffolds, etc) can be obtained from the corresponding authors upon reasonable request.

April 2023

Research involving human participants, their data, or biological material

Policy information about studies with human participants or human data. See also policy information about sex, gender (identity/presentation), and sexual orientation and race, ethnicity and racism.

Reporting on sex and gender	N/A
Reporting on race, ethnicity, or other socially relevant groupings	N/A
Population characteristics	N/A
Recruitment	N/A
Ethics oversight	N/A

Note that full information on the approval of the study protocol must also be provided in the manuscript.

Field-specific reporting

Please select the one below that is the best fit for your research. If you are not sure, read the appropriate sections before making your selection.

☒ Life sciences ☐ Behavioural & social sciences ☐ Ecological, evolutionary & environmental sciences

For a reference copy of the document with all sections, see nature.com/documents/nr-reporting-summary-flat.pdf

Life sciences study design

All studies must disclose on these points even when the disclosure is negative.

Sample size	This was the first study in which biodegradable tissue engineered heart valves were implanted in the aortic position, therefore no relevant literature was available that could be used for sample size calculation. However, our research group does have vast experience with similar heart valves implanted in the pulmonary position. Although direct translation of the results obtained in pulmonary position to aortic position is not relevant for an accurate sample size calculation, we can use our experience to make a estimate. Based on our previous study with pulmonary tissue engineered heart valves (Kluin et al 2017, biomaterials), we found that a total group size of n=20 animals is sufficient to study the functionality and tissue formation on the valves.
Data exclusions	We lost n=5 animals due to surgical complications. The specific reasons of death for each animal that was excluded is described in the results section of the manuscript.
Replication	This is the first study with biodegradable tissue engineered aortic heart valve prostheses. We have not replicated any earlier data.
Randomization	We have randomly allocated all animals for their follow-up period. This was done by computer randomization, executed by an independent researcher not involved in this project.
Blinding	All animals received the same type of heart valve/intervention, therefore blinding was not needed/implemented.

Reporting for specific materials, systems and methods

We require information from authors about some types of materials, experimental systems and methods used in many studies. Here, indicate whether each material, system or method listed is relevant to your study. If you are not sure if a list item applies to your research, read the appropriate section before selecting a response.

Materials & experimental systems

n/a	Involved in the study	
☒	☐	Antibodies
☒	☐	Eukaryotic cell lines
☒	☐	Palaeontology and archaeology
☐	☒	Animals and other organisms
☒	☐	Clinical data
☒	☐	Dual use research of concern
☒	☐	Plants

Methods

n/a	Involved in the study	
☒	☐	ChIP-seq
☒	☐	Flow cytometry
☒	☐	MRI-based neuroimaging

Animals and other research organisms

Policy information about studies involving animals; ARRIVE guidelines recommended for reporting animal research, and Sex and Gender in Research

Laboratory animals	Swifter sheep, 2 years old
Wild animals	No wild animals were used
Reporting on sex	Only female sheep were used. We used only female sheep to improve animal welfare during housing. By choosing female sheep only, the animals could be socially housed in groups. The use of both males and females would disturb the peace and cause distress in the stable and animals should have been housed separately.
Field-collected samples	No samples were collected from the field.
Ethics oversight	Approval of the animal studies was obtained by the Amsterdam University Medical Centers Animal Care Ethics Committee (AVD1180020197705) and are in agreement with the current Dutch law on animal experiments (WOD).

Note that full information on the approval of the study protocol must also be provided in the manuscript.
